# Comparing the Efficacy of Antiosteoporotic Drugs in Preventing Periprosthetic Bone Loss Following Total Hip Arthroplasty: A Systematic Review and Bayesian Network Meta‐Analysis

**DOI:** 10.1111/os.14165

**Published:** 2024-07-26

**Authors:** Yi Tang, Zhaokai Jin, Yichen Lu, Lei Chen, Shuaijie Lv, Taotao Xu, Peijian Tong, Guoqian Chen

**Affiliations:** ^1^ The First Affiliated Hospital of Zhejiang Chinese Medical University (Zhejiang Provincial Hospital of Chinese Medicine) Zhejiang China

**Keywords:** antiosteoporotic drugs, bone loss, network Meta‐analysis, THA

## Abstract

**Background:**

Periprosthetic bone loss is a well‐known phenomenon following total hip arthroplasty (THA). However, the choice of drugs for prevention remains controversial. Therefore, the aim of this study was to determine the best drug to treat periprosthetic bone loss by comparing changes in bone mineral density (BMD) at different times after THA.

**Methods:**

A comprehensive search of five databases and two clinical trial registration platforms was undertaken from their inception through to August 31, 2023 to identify eligible randomized controlled trials. A Bayesian network meta‐analysis (NMA) was carried out for calculating the standardized mean difference (SMD) and the surface under cumulative ranking curve (SUCRA) of the BMD in calcar (Gruen zone 7) at 6 months, 12 months, and 24 months and over.

**Results:**

Twenty‐nine trials involving 1427 patients and 10 different interventions were included. The results demonstrated that at 6 months, denosumab had the highest ranking (SUCRA = 0.90), followed by alendronate (SUCRA = 0.76), and zoledronate (SUCRA = 0.73). At 12 months, clodronate ranked highest (SUCRA = 0.96), followed by denosumab (SUCRA = 0.84) and teriparatide (SUCRA = 0.82). For interventions with a duration of 24 months and over, denosumab had the highest SUCRA value (SUCRA = 0.96), followed by raloxifene (SUCRA = 0.90) and zoledronate (SUCRA = 0.75).

**Conclusion:**

Investigating the existing body of evidence revealed that denosumab demonstrates potential as an intervention of superior efficacy at the three specifically examined time points. However, it remains crucial to conduct further research to confirm these findings and determine the most effective treatment strategy.

## Graphical abstract

Based on a network meta‐analysis of randomized controlled trials, we conclude that denosumab is the most effective drug for the prevention of periprosthetic osteoporosis after total hip arthroplasty.

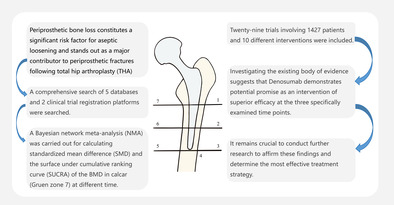

## Introduction

**FIGURE 1:**
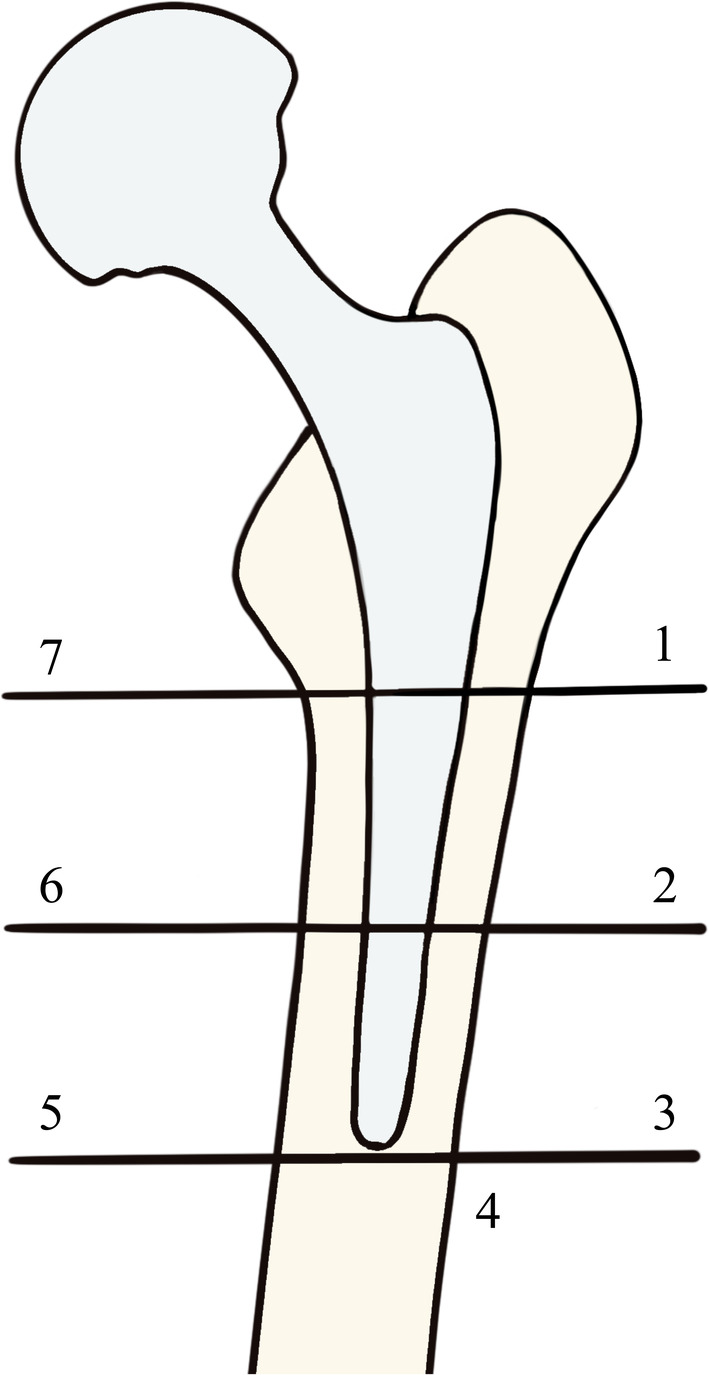
Schematic diagram of Gruen zones. Zone 7 represents the medial region above the lesser trochanter.

According to the 2023 American Joint Replacement Registry (AJRR) annual report,^1^ the United States witnessed a total of 95,495 revision hip arthroplasty procedures between 2012 and 2022. Of these, 20.74% were caused by instability‐related codes, 19.17% by aseptic loosening, 14.02% by facture, and 6.94% by wear or osteolysis. Periprosthetic bone loss plays a significant role in this process, making it crucial to address this issue proactively in revision hip arthroplasty. The introduction of a prosthesis reduces the stress that would otherwise be borne by the bone, resulting in localized bone resorption, a phenomenon known as stress shielding.^2^ Notably, the medial region above the lesser trochanter, specifically Gruen zone 7 (Figure 1),^3^ exhibits the most substantial bone loss, as observed in bone mineral density (BMD) measurements.^4^ Consequently, the prevention of bone loss emerges as a pivotal concern in clinical practice following total hip arthroplasty (THA), particularly in Gruen zone 7.

The bone loss caused by stress shielding is difficult to solve through the design of the prosthesis. Typically, researchers aspire to address this issue by using anti‐osteoporosis drugs. Previous pairwise meta‐analysis has hinted at the potential benefits of antiosteoporotic drugs^5^ in mitigating bone loss subsequent to THA. Nevertheless, these analyses are limited in their simultaneous comparison of multiple interventions. Addressing this limitation, network meta‐analysis (NMA) provides a statistical framework enabling the comprehensive evaluation of multiple interventions through both direct and indirect comparisons. A prior NMA, incorporating 21 clinical studies, compared the effectiveness of nine antiosteoporotic drugs in preventing bone loss.^6^ However, the study did not include more recent drugs, such as raloxifene. At the same time, it lacked a comprehensive exploration of the underlying factors that affect the efficacy. It also ignored the long‐term efficacy of drugs, which is a prominent issue for osteoporosis patients. The existence of these shortcomings means there is a possibility of biased results.

Therefore, our study aims to conduct a thorough NMA, systematically comparing the efficacy of antiosteoporotic drugs in averting periprosthetic bone loss following THA. The outcomes of this research have the potential to enhance clinical practice by equipping clinicians with more effective strategies to prevent bone loss and its associated complications following THA.

## Methods

This study followed the Preferred Reporting Items for Systematic Reviews and Meta‐Analyses for Network Meta‐Analyses (PRISMA‐NMA).^7^ This study protocol was registered at PROSPERO under the number CRD42023443963.

### Literature Search

To comprehensively search for qualified trials, PubMed, Embase, Scopus, Cochrane Library, and Web of Science were searched from the inception of each database to August 31, 2023. The retrieval strategies incorporated medical subject headings terms (e.g., MeSH for PubMed), keywords, and free terms. Reference lists of the review articles were also explored. In addition, a gray literature search was conducted to find clinical trials with unpublished data on the ClinicalTrials.gov and the International Clinical Trial Registration Platform (ICTRP). Primary investigators of any identified ongoing trials were contacted by email to request the latest data.

### Inclusion and Exclusion Criteria

Population, Interventions, Comparisons, Outcomes and Study design (PICOS)^8^ as a framework to formulate eligibility criteria of the included studies in our NMA were carefully designed as follows:

1. Population: The eligible population in this NMA where patients underwent primary THA without any restrictions on sex, age, or disease duration. Moreover, eligibility criteria of participants must be reported in the original study.

2. Intervention: The eligible types of intervention in the experimental group could be a single antiosteoporotic drug, a combination of two or more antiosteoporotic drugs, or an antiosteoporotic drug plus other active drug.

3. Comparisons: Eligible types of comparators in the control group could be different antiosteoporotic medications from the treatment group and any inactive interventions, such as blank controls and placebo.

4. Outcomes: The outcome of this NMA was periprosthetic bone loss in calcar (zone 7). Specifically, it was measured by the percentage increase or decrease in BMD relative to baseline.

5. Study designs: For this NMA, only randomized controlled trials (RCTs) were eligible.

Studies that meet the following criteria were excluded: (i) studies conducted with non‐anti‐osteoporosis drugs; (ii) animal experiments or cell experiments; (iii) non‐RCTs; (iv) studies with missing and unavailable data; and (v) research published in a language other than English.^9^

### Study Selection and Data Extraction

The retrieval records obtained from databases were imported into Zotero (Corporation for Digital Scholarship) for study selection by two researchers. After removing duplicate studies, the titles and abstracts were reviewed by two investigators to determine their eligibility. Final inclusion was determined after screening the full text of the remaining articles. In addition, we searched the reference lists of the eligible studies to identify any other RCTs that met our inclusion criteria. In case of any disagreements between the two researchers during the study selection process, a senior investigator was consulted to resolve the discrepancies. Two investigators independently used Microsoft Excel (Microsoft, Redmond, Washington, USA) to extract data from the included RCTs. The extracted information and data encompassed five primary categories, containing basic study details (first author, publication year, and article title), participant information (sample size, sex distribution, and average age), intervention details (antiosteoporotic drugs and type of prosthesis), methods information (study design and time points of outcome measures), and outcomes. All data entered in Excel were double‐checked and verified by a third researcher to ensure consistency and validity. We extracted the required data according to the *Cochrane Handbook for Systematic Reviews of Interventions* version 6.4 (updated August 2023)^10^ and transformed it into the necessary format. When only figures were presented, we used Origin software (OriginLab, Northampton, MA, USA) to measure the pixel length of the axes for calibration. Subsequently, we determined the pixel length from the relevant axis to the data points of interest. If any crucial data were missing, we contacted the corresponding authors by email to request the necessary information. In the case of three‐arm studies, data from these studies was split and processed in the pairwise meta‐analysis, which is a widely recognized method.^11^ The kappa agreement index was used to evaluate the level of agreement between the two reviewers.^12^ The degree of consistency was identified as slight, fair, moderate, substantial, and almost perfect when the kappa coefficients were calculated as 0.00–0.20, 0.21–0.40, 0.41–0.60, 0.61–0.80, and 0.81–1.00, respectively.

### Geometry of the Network and Summary Measures

Before performing this NMA, we assessed the transitivity assumption, which requires studies comparing different sets of interventions to be sufficiently similar to provide valid indirect inferences. We used R (R Foundation for Statistical Computing, Vienna, Austria) software, particularly the GeMTC package, to conduct NMA, and the ggplot2 package for graphic rendering. Standardized mean differences (SMDs) were converted into relative format and entered into the GeMTC package for analysis.^13^

Network plots were created to visually illustrate the comparative relationships among different interventions. Each intervention was depicted as a node, with the size of the node indicating the sample size used to assess that particular intervention. The lines connecting the nodes in the network plots represented direct comparisons between two interventions, with thicker lines indicating a higher number of direct comparisons. League tables were presented with summary SMDs and 95% credible intervals for all pairwise comparisons. The forest plot presented the results comparing each intervention group's outcomes with the control group. In addition, we obtained a hierarchy of competing interventions using the surface under the cumulative ranking curve (SUCRA). SUCRA values were expressed as a percentage, indicating the relative probability of an intervention being among the best options. A higher value represents a higher probability of being the best option.

### Risk of Bias within Individual Studies

The methodological quality of each RCT included in the study was independently assessed by two raters using the Cochrane Collaboration's risk of bias tool (version 2.0, RoB 2).^14^ The risk of bias for each included RCT was evaluated based on five critical factors: the randomization process, deviations from the intended interventions, missing outcome data, outcome measurements, and choice of reported outcomes. Each of these domains was classified as having a low, unclear, or high risk of bias. In cases of disagreement between the two raters, a referee was involved to mediate and resolve the dispute. The kappa coefficient was calculated to identify the consistency between two reviewers on the methodological quality assessment of each study.

### Assessment of Inconsistency and Risk of Bias across Studies

The *I* ^2^‐parameter was used to quantify heterogeneity between the results of each study. If *I* ^2^ >50%, this indicates substantial heterogeneity. We will explore potential reasons for heterogeneity using network meta‐regression. We also performed a statistical evaluation of consistency, which comprised agreement between direct and indirect evidence. If primary outcomes were reported in 10 or more included studies, we would use funnel plots to evaluate publication bias. Additionally, we assessed the effects of significant publication bias on meta‐analysis outcomes by conducting Egger's tests, where a *p*‐value less than 0.05 indicated significant bias.

### Assessment of Quality of Evidence

We used Confidence in Network Meta‐Analysis (CINeMA) to assess the plausibility of the results. It is based on a methodological framework including six domains: within‐study bias, reporting bias, indirectness, imprecision, heterogeneity, and incoherence.^15^, ^16^

### Additional Analyses

To ensure the comprehensiveness of the comparison, we performed additional analyses grouped by drug mechanism to discuss the effects of different mechanisms of action on efficacy.

## Results

### Study Selection and Study Characteristics

**FIGURE 2:**
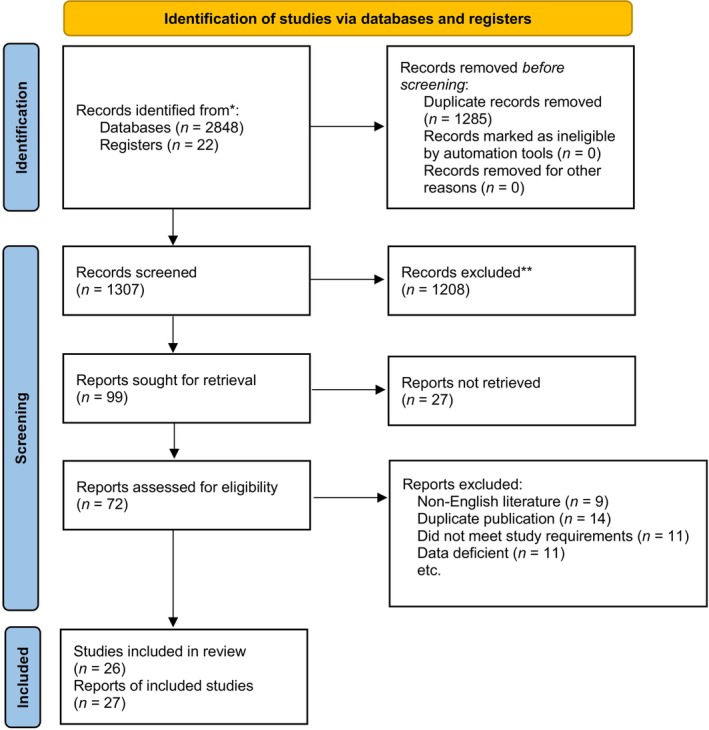
Preferred Reporting Items for Systematic Reviews and Meta‐Analyses (PRISMA) 2020 flow diagram for systematic reviews.

**FIGURE 3:**
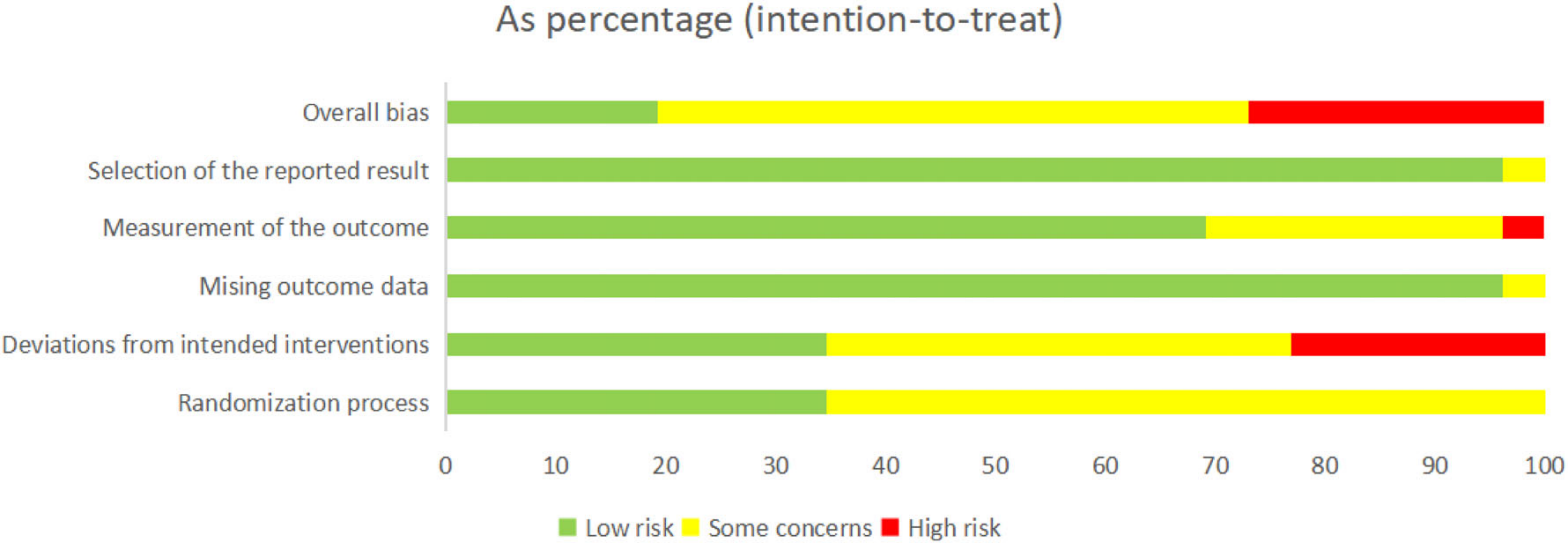
Summary of the risk of bias assessment in the individual domains of the included studies.

Following the previously described research strategy (Appendix 1), 27 studies were included in the review,^17^, ^18^, ^19^, ^20^, ^21^, ^22^, ^23^, ^24^, ^25^, ^26^, ^27^, ^28^, ^29^, ^30^, ^31^, ^32^, ^33^, ^34^, ^35^, ^36^, ^37^, ^38^, ^39^, ^40^, ^41^, ^42^, ^43^, ^44^ 26 of which were used for analysis^17^, ^18^, ^19^, ^20^, ^21^, ^22^, ^23^, ^24^, ^25^, ^26^, ^27^, ^28^, ^29^, ^30^, ^31^, ^32^, ^33^, ^34^, ^35^, ^36^, ^37^, ^38^, ^39^, ^40^, ^41^, ^42^, ^43^ (Appendix 2), with 1427 patients enrolled. The kappa inter‐rater reliability score of title/abstract screening and full‐text reading were 0.81 (*p* < 0.001) and 0.89 (*p* < 0.001). One study^44^ met the other requirements but was included in the review without analysis because it did not report changes in BMD in the form of SMD. A total of 24 analyzed studies used uncemented prostheses, and only two studies used cemented prostheses. The mean age of the enrolled patients was approximately 63 years, and the sex ratio (male/female) was 32.6%. The longest follow‐up time was 9 years. The screening procedure is shown in Fig. 2. The risk of bias assessments for each individual study (Appendix 3) are summarized in Fig. 3. The kappa coefficients ranged from 0.85 (*p* < 0.001) to 0.92 (*p* < 0.001), indicating that the consistency of two reviewers’ assessment on the quality of each study was almost perfect. Table 1 shows the characteristics of the included studies. After convergence evaluation, we determined that the model could achieve reasonable convergence results when processing the data (Appendix 4). The results of the NMA are presented in league tables (Appendix 5).

**TABLE 1:** Characteristics of the included studies

Studies	Follow‐up time (year)	Sample size	Mean age	Sex radio (man/woman)	Mean BMI	Prosthesis type
Arabmotlagh et al. (2006)	1	51	62.53	1.04	28.91	Uncemented
Arabmotlagh *et al*. (2009)	6	49	60.41	1.04	‐	Uncemented
Aro *et al*. (2018)	4	49	68.09	0	29.09	Uncemented
Finnilä *et al*. (2021)	2	57	68.83	0	27.98	Uncemented
Fokter *et al*. (2005)	1	46	70	0.31	27.18	Cemented
Fokter *et al*. (2006)	1	31	68.42	0.41	28.05	Cemented
Gong *et al*. (2020)	2	240	62.85	0	26.9	Uncemented
Huang *et al*. (2017)	2	54	59.75	1.16	25.5	Uncemented
Iwamoto *et al*. (2011)	2	54	72.22	0.3	27	Uncemented
Iwamoto *et al*. (2014)	2	60	64.7	3.92	24.34	Uncemented
Kobayashi *et al*. (2016)	1	46	65.35	0.15	23.42	Uncemented
Morita *et al*. (2020)	2	47	64.87	0.15	23.43	Uncemented
Nagoya *et al*. (2018)	1	20	79.6	0	‐	Uncemented
Nakura *et al*. (2023)	2	82	69.08	0.06	‐	Uncemented
Nishioka *et al*. (2007)	1	15	71.73	0.06	‐	Uncemented
Scott *et al*. (2013)	2	51	61.2	0.82	29.1	Uncemented
Sköldenberg *et al*. (2011)	2	73	60.49	1.43	27.51	Uncemented
Tapaninen *et al*. (2010)	5	16	61.63	0.78	28.11	Uncemented
Trevisan *et al*. (2010)	1	91	64.72	1.39	27.02	Uncemented
Venesmaa *et al*. (2001)	0.5	13	62.62	0.86	26.99	Uncemented
Yamaguchi *et al*. (2003)	1	52	14.52	0.27	‐	Uncemented
Yamaguchi *et al*. (2004)	2	55	66.02	0.15	‐	Uncemented
Yamaguchi *et al*. (2005)	1	43	68.09	0	‐	Uncemented
Yamasaki *et al*. (2007)	0.5	40	66.75	0.11	‐	Uncemented
Yukizawa *et al*. (2017)	9	60	48.77	0.39	‐	Uncemented
Zhou *et al*. (2019)	1	32	73.85	0	24.15	Uncemented

Abbreviation: BMI, body mass index.

### Synthesis of Results

**FIGURE 4:**
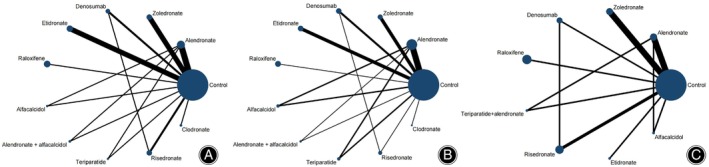
Network plots at different times. The plots (A), (B), and (C) represent the network plots at 6, 12, and 24 months and over, respectively. Each dot represents a different intervention, with larger dots representing that a larger sample size was included. Lines represent direct comparisons, with thicker lines indicating more studies that performed direct comparisons.

**FIGURE 5:**
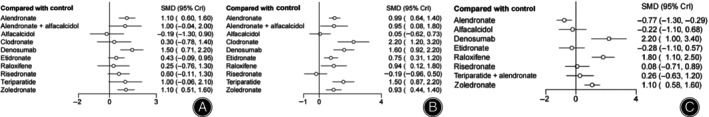
Forest plots at different times. The plots (A), (B), and (C) represent the forest plots at 6, 12, and 24 months and over, respectively. Forest plots were used to represent the treatment effect of each intervention relative to the control group. CrI, credible intervals; SMD, standardized mean difference.

A total of 23 studies with 1272 samples compared 10 different interventions with a control group at 6 months (Figure 4A). Compared with the control group, we found alendronate (SMD 1.11, 95% confidence interval [CI] 0.60 to 1.64), denosumab (SMD 1.46; 95% CI 0.71 to 2.17), and zoledronate (SMD 1.05; 95% CI 0.51 to 1.60) showed significant efficacy (Figure 5A). Denosumab had the highest ranking (SUCRA = 0.90), followed by alendronate (SUCRA = 0.77) and zoledronate (SUCRA = 0.73) (see Table 2). Among the three effective therapies, denosumab has a greater therapeutic effect than alendronate and zoledronate.

**TABLE 2:** SUCRA of prophylactic efficacy on BMD in zone 7

Treatment	6 months	12 months	24 months and over
Alendronate	0.76	0.56	0.04
Alendronate + alfacalcidol	0.68	0.54	NA
Alfacalcidol	0.13	0.14	0.28
Clodronate	0.33	0.96	NA
Control	0.14	0.12	0.39
Denosumab	0.90	0.84	0.96
Etidronate	0.38	0.41	0.25
Raloxifene	0.30	0.53	0.90
Risedronate	0.47	0.07	0.43
Teriparatide	0.68	0.82	NA
Teriparatide + alendronate	NA	NA	0.51
Zoledronate	0.73	0.52	0.75

Abbreviations: BMD, bone mineral density; NA, not applicable; SUCRA, surface under cumulative ranking curve.

At 12 months, 27 studies with 1328 samples compared 10 different interventions with the control group (Figure 4B). The forest plot indicates that, except for alfacalcidol (SMD 0.05; 95% CI −0.62 to 0.73) and risedronate (SMD −0.19; 95% CI −0.96 to 0.50), other treatment interventions have relatively reliable therapeutic effects (Figure 5B). Among them, clodronate (SMD 2.20; 95% CI 1.20 to 3.20), denosumab (SMD 1.60; 95% CI 0.92 to 2.20), and teriparatide (SMD 1.50; 95% CI 0.87 to 2.20) exhibit significant advantages. Table 2 shows that clodronate (SUCRA = 0.96) had the best efficacy, while denosumab (SUCRA = 0.84) and teriparatide (SUCRA = 0.82) had similar effects but still had a noticeable difference compared to clodronate.

When the time was equal to or more than 24 months, 11 studies with 756 samples compared eight different interventions with the control group (Figure 4C). Among all the interventions, denosumab (SMD 2.20; 95% CI 1.10 to 3.40), raloxifene (SMD 1.80; 95% CI 1.10 to 2.50), and zoledronate (SMD 1.10; 95% Cl 0.58 to 1.60) showed a significant improvement in treatment effect (Figure 5C). In addition, alendronate showed a certain negative effect (SMD −0.77; 95% CI −1.30 to −0.29). In the comparison of SUCRA (Table 2), denosumab had the best effect (SUCRA = 0.96), followed by raloxifene (SUCRA = 0.90) and zoledronate (SUCRA = 0.75).

### Heterogeneity and Certainty of Evidence

A heterogeneity test showed that there was some heterogeneity at 6 months and 12 months, and the heterogeneity was not obvious at 24 months and over (Table 3). The differences of deviance information criterion (DIC) in each group were small, so the data were basically in accordance with global consistency. The Side test of all outcomes showed that the percentage of comparisons with evidence of inconsistency ranged from 16.7% to 21.7%. Local inconsistencies exist in the direct and indirect comparisons between risedronate and denosumab at 6 months and 12 months and between alendronate and alfacalcidol at 24 months and over (Appendix 6).

**TABLE 3:** Evaluation of heterogeneity and inconsistency

Outcomes	Number of studies	Heterogeneity		DIC		Side splitting	
		i2.pair	i2.cons	Consistency model	Inconsistency model	Number of inconsistent comparisons out of total	Percentage of inconsistent comparisons out of total
6	23	9.77	57.11	45.6	40.1	5	21.7%
12	24	2.55	38.36	48.8	45	4	16.7%
24	11	<0.001	<0.001	21.0	21	2	18.2%

**FIGURE 6:**
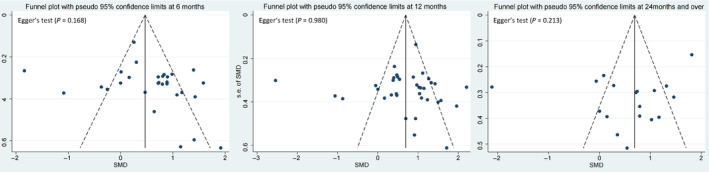
Funnel plots at different times. SMD, standardized mean difference.

The potential threats posed by the baseline characteristics of the included studies to the source of heterogeneity were addressed through comprehensive meta‐regression conducted for all outcomes (Table 4). Sensitivity analyses that excluded studies with a high overall risk of bias and studies using cemented prostheses, respectively, confirmed the robustness of our previous study (Appendix 7). We also evaluated the primary outcome of publication bias. The funnel plots showed no evidence of asymmetry, and the results of Egger's test showed that no small study effect was found (Fig. 6). Therefore, the outcomes were deemed to have no publication bias.

**TABLE 4:** Network meta‐regression

Covariate	Shared beta (median and 95% CrI)		
	6 month	12 month	24 month and over
None	‐	‐	‐
Publication year	−0.48 (−1.27, 0.32)	−0.10 (−0.91, 0.68)	0.11 (−1.15, 1.37)
Follow‐up years	0.04 (−0.56, 0.68)	−0.08 (−0.52, 0.36)	−0.07 (−1.34, 1.39)
Sample size	0.10 (−1.20, 1.45)	0.34 (−1.02, 1.59)	−0.00 (−4.28, 3.3)
Mean age	−0.33 (−0.81, 0.19)	−0.34 (−0.73, 0.04)	−0.16 (−1.54, 1.16)
Mean BMI	0.02 (−0.53, 0.56)	−0.25 (−0.67, 0.19)	0.05 (−1.18, 1.26)
Sex ratio	0.09 (−0.48, 0.64)	0.11 (−0.34, 0.56)	0.74 (−0.60, 2.03)
Prosthesis type	0.20 (−0.83, 1.26)	0.51 (−0.35, 1.40)	0.79 (−0.47, 2.07)

Abbreviations: BMI, body mass index; CrI, credible intervals.

Using the CINeMA method, we assessed the confidence of the findings (Appendix 8). The level of evidence in these studies was generally moderate to high. There are some concerns and uncertainties regarding the different aspects of the comparison; these concerns and uncertainties mainly focus on within‐study bias and incoherence. However, most comparisons received high ratings and had relatively stable results across studies over time.

### Results of Additional Analyses

Different interventions were grouped according to pharmacology and mechanism of action for additional analyses (Appendix 9). RANKL inhibitors had the best effects across studies over time, and the advantage was significant. The bisphosphonates (BPs) group showed notably better treatment outcomes than the control group at 6 and 12 months. The PTHrP group showed a better treatment effect than the control group only at 12 months. Differences in treatment effects in the other groups did not reach significance.

## Discussion

Our study highlights the varying efficacy of different interventions over different times with a Bayesian NMA. At 6 months, denosumab, alendronate, and zoledronate showed the highest degree of efficacy when compared to the control group. At 12 months, most drugs achieved positive effects, with clodronate, denosumab, and teriparatide showing advantages. At 24 months and over, denosumab, raloxifene, and zoledronate were the most effective treatments. Denosumab consistently demonstrated reliable efficacy at most time points, suggesting it might be a particularly effective treatment option.

Periprosthetic bone loss constitutes a significant risk factor for aseptic loosening^45^ and stands out as a major contributor to periprosthetic fractures following THA.^46^ Notably, THA revision surgeries, prompted by such issues,^47^ entail a heightened risk of both local and systemic complications.^48^ Periprosthetic bone loss plays an important role in this process. Therefore, a strategic focus on mitigating revision hip arthroplasty involves proactively addressing bone loss around the prosthesis. The introduction of a prosthesis results in a reduction of stress that would otherwise be borne exclusively by the bone, leading to localized bone resorption, a phenomenon termed stress shielding.^2^ The stiff stem of a prosthesis demonstrates a significantly higher incidence of pronounced bone resorption due to stress shielding compared to the flexible stem. Although the flexible stem allows for greater relative motion between the stem and the femoral bone, this micromotion can contribute to late stem loosening, attributed to fibrous fixation and the development of osteolytic foci.^25^ Given these intricacies, achieving the ideal prosthesis is a formidable challenge. Instead, a more pragmatic approach involves the prevention of complications through pharmacological intervention.

Denosumab is a monoclonal antibody to the receptor activator of nuclear factor‐kB ligand (RANKL),^49^ a key regulator of bone resorption through its effect on osteoclast development, function, and survival.^50^ Research has shown that long‐term use of denosumab treatment can lead to a sustained increase in bone density for up to 10 years while maintaining a low incidence of adverse events.^51^ Nystrom *et al*.^44^ found that injection of denosumab after TKA resulted in a rapid and systemic reduction in biochemical markers of bone resorption and effective preservation of periprosthetic BMD. This effect was lost by 18 months after the last denosumab injection. In our study, both the SUCRA ranking of denosumab in the comparison of different interventions and the SUCRA ranking of RANKL receptor inhibitors in the comparison of drugs with different mechanisms have obvious advantages. Therefore, we believe that denosumab might have the best effect in preventing periprosthetic bone loss after THA.

In addition to denosumab, other interventions showed positive effects at various time points. Nevertheless, the formulation of treatment strategies must consider more than theoretical efficacy. The effectiveness of diverse interventions can differ based on individual patient characteristics and preferences. Other factors, including potential side effects and cost, should be considered. Alendronate, clodronate, and zoledronate are BPs that reduce bone loss by inhibiting bone resorption.^52^ However, the effectiveness of alendronate diminishes over time. In our study, a negative effect of alendronate was observed when the duration was longer than 24 months. This is not consistent with the common perception. In addition to drug tolerance and reconstruction of bone balance, we speculate that it might be related to the longer follow‐up time. The compliance of patients with long‐term use of alendronate is not as good as that for other drugs, and older age also affects the efficacy.^53^ One study we included reported a decrease in BMD around the prosthesis and an increase in the lumbar spine.^5^ This might indicate that the negative effects of alendronate are only present at specific sites, especially in areas where stress shielding occurs. Clodronate is more effective at 12 months compared to 6 months, potentially because it has a higher rate of absorption.^54^ Due to the lack of long‐term studies, this possibility has not been fully demonstrated. Zoledronate, being a long‐acting bisphosphonate, consistently exhibits stable effects most of the time, which is attributed to its unique mechanism of inhibiting bone resorption.^55^ Previous studies have shown that in postmenopausal osteoporosis patients who had been on long‐term BP therapy, switching to denosumab was associated with greater gains than zoledronate,^56^ which also supports the better efficacy of denosumab. In contrast, risedronate, which also belongs to the BP group, did not have a satisfactory effect. Although BPs are safe drugs in most cases, they might also produce side effects such as gastrointestinal disorders, arthralgia, and elevated erythrocyte sedimentation rate and C‐reactive protein,^57^ which might limit their use in certain populations. Additionally, teriparatide and raloxifene have shown interesting effects. Teriparatide acts on the PTH receptor 1, inducing a marked increase in bone formation markers and simultaneously inhibiting bone loss caused by sustained elevation of parathyroid hormone levels.^58^ A meta‐analysis^59^ has shown that teriparatide might be superior to alendronate in improving lumbar BMD in postmenopausal osteoporosis patients. However, teriparatide is relatively less recommended compared to drugs such as denosumab due to its higher cost,^60^ and teriparatide is associated with a risk of rapid loss of BMD after discontinuation.^40^ Raloxifene, a second‐generation selective estrogen receptor modulator (SERM), can bind to estrogen receptors and produce estrogen‐like effects on the bones, reducing absorption and increasing BMD in postmenopausal women.^61^ There might be a delayed effect of raloxifene,^62^ which could explain why raloxifene showed a positive effect in this study at 24 months and over. Raloxifene is commonly used in clinical practice for women, while denosumab exhibits broader applicability. Furthermore, denosumab is superior to raloxifene in reducing the risk of death and ischemic stroke in women with osteoporosis.^63^ Overall, compared with interventions, denosumab has a higher priority when multiple factors are considered.

While striving for consistent results in research, it is essential to acknowledge the potential variations among different studies, as these variations can significantly influence the outcomes. Therefore, meticulous interpretation and inference of study results are necessary, considering the presence of heterogeneity and inconsistencies when evaluating the evidence. In our study, heterogeneity was primarily observed at 6 months and 12 months. Despite conducting a meta‐regression analysis of baseline characteristics, no potential sources of heterogeneity were identified, indicating the contribution of unknown factors to the heterogeneity of results. In more long‐term studies, heterogeneity was almost completely eliminated. In examining consistency, we noted that at 6 months and 12 months, denosumab was superior in the direct comparison, but risedronate was superior in the indirect comparison. Slight inconsistencies were noted in the comparison of alendronate and alfacalcidol at 24 months and beyond. Different studies might have different biases and random errors, which might lead to inconsistent results. Considering the degree of credibility of the study results derived from the CINeMA method, we believe that the results of the direct comparison of denosumab are more credible. Neither alendronate nor alfacalcidol had reliable effects in long‐term studies and, thus, did not affect our conclusions. Overall heterogeneity and inconsistency were within acceptable limits.

Shi et al.^5^ conducted a meta‐analysis to assess the effectiveness of BPs in preventing bone loss after THA, finding that third‐generation BP, in particular, can prevent bone loss and might be effective in averting skeletal complications following THA. Nevertheless, the authors note that more research is required to fully comprehend the possible side effects of BP usage. Li *et al*.^64^ found through meta‐analysis that both zoledronate and denosumab were efficacious in reducing bone loss and promoting patient recovery after THA. These treatments not only enhance bone density but also modify bone metabolism markers, ultimately leading to improved assessment results for patients undergoing THA. Liu *et al*.^65^ evaluated the effectiveness of zoledronate in treating bone loss after THA and found that it maintains BMD and improves hip joint function. However, the study also acknowledges the potential side effect of jawbone necrosis. Thus, zoledronate can be recommended as an alternate or supplementary therapy to traditional surgical or drug therapies. According to Yang's NMA,^66^ denosumab is the top‐ranked antiosteoporotic drug for enhancing total hip BMD in postmenopausal women, followed by zoledronate and teriparatide. This finding is consistent with the results of our own study, which also found denosumab to be the most effective treatment for improving total hip BMD. Chen *et al*. found that denosumab had the best efficacy at 12 months, but their study lacked data on denosumab at 6 months and longer‐term research on all drugs.^6^ While the highest rate of periprosthetic BMD loss occurs within the first year following THA,^67^ it is important to note that bone loss continues for up to three decades after surgery.^68^ Given that prostheses usually have a long useful life, a long follow‐up is warranted. Our study, conducted through a comprehensive literature search, effectively addresses this identified data void at the 6‐months interval for denosumab, affirming its superior efficacy, which is a distinct strength of our study.

Our study has several limitations. Almost all the identified comparative evidence is focused on BPs, with very few closed‐loop comparisons among other active drugs. Therefore, the lack of indirect comparisons might introduce bias. Some drugs have been proven effective in treating osteoporosis, such as abaloparatide^69^ for stimulating bone formation and romosozumab,^70^ with anti‐resorptive and anabolic properties. However, they were excluded from our study due to not meeting the inclusion criteria, potentially causing us to miss out on some effective medications. Furthermore, studying the effects of combined and personalized treatment approaches might provide valuable insights into optimizing the management of bone loss, which could also be one of the main directions of future exploration.

## Conclusion

In conclusion, this NMA confirms that antiosteoporotic drugs have clear benefits in preventing periprosthetic bone loss following THA. Denosumab appears to be a promising intervention with potentially superior efficacy across the three time points analyzed. After statistical analysis and discussion, the results of our study can basically be considered robust, although some limitations remain. Future studies should aim to validate these findings by using larger sample sizes and standardized research protocols, thus enabling more precise evaluations of treatment efficacy.

## Conflict of Interest Statement

The author has no conflicts of interest to declare.

## Ethics Statement

Ethics approval and consent are not applicable in this study.

## Author Contributions

Yi Tang collected and analyzed data and drafted the manuscript. Yi Tangyi, Zhaokai Jin and YIchen Lu performed the statistical analysis. Yi Tang, Lei Chen, Shuaijie Lv and Taotao Xu interpreted the data. All listed authors have each made substantial contributions to conception and design, acquisition of data, or analysis and interpretation of data. Peijian Tong and Guoqian Chen designed the project and participated in revising it critically for content, and have approved the final version of the submitted manuscript.

## Supporting information


**Data S1.** Supporting information.
