# Supplementary material for: Comparing the Efficacy of Antiosteoporotic Drugs in Preventing Periprosthetic Bone Loss Following Total Hip Arthroplasty: A Systematic Review and Bayesian Network Meta‐Analysis
Source: Orthop Surg. 2024 Jul 26;16(10):2344–54. doi: 10.1111/os.14165 (PMC11456730; doi:10.1111/os.14165)
Supplement: Supplementary file 1 — Data S1. Supporting information. [file OS-16-2344-s001.docx]

**Supplementary Material**

**Catalogue**

[Appendix 1: Search strategy 3](#_Toc11166)

[Appendix 2: Characteristics of studies and subjects included in the NMA 5](#_Toc22497)

[Table 2.1 Characteristics of eligible RCTs included in NMA 5](#_Toc25071)

[Table 2.2 Data in NMA (6months) 12](#_Toc5748)

[Table 2.3 Data in NMA (12months) 13](#_Toc17228)

[Table 2.4 Data in NMA (24months and over) 14](#_Toc22080)

[List of included studies 16](#_Toc9951)

[Appendix 3: The risk of bias assessment for the individual included studies. 20](#_Toc22212)

[Figure 3.1 ROB2 figure (ITT) 20](#_Toc26427)

[Appendix 4: Diagnosis of convergence 21](#_Toc1294)

[Figure 4.1 Gelman plot (6 months) 21](#_Toc28384)

[Figure 4.2 Gelman plot (12 months) 22](#_Toc5308)

[Figure 4.3 Gelman plot (24 months and over) 23](#_Toc12703)

[Appendix 5 NMA result 24](#_Toc17987)

[Figure 5.1: Individual study results grouped by treatment comparison (6 months) 24](#_Toc16990)

[Figure 5.2: Individual study results grouped by treatment comparison (12 months) 25](#_Toc27692)

[Figure 5.3: Individual study results grouped by treatment comparison (24 months) 26](#_Toc28626)

[Table 5.1: League Table (6 months) 27](#_Toc3936)

[Table 5.2: League Table (12 months) 28](#_Toc26659)

[Table 5.3: League Table (24 months and over) 29](#_Toc21187)

[Appendix 6: Results of SIDE splitting results 30](#_Toc8857)

[Table 6.1 Results of SIDE splitting results (6 months) 30](#_Toc30886)

[Table 6.2 Details of SIDE splitting results (12 months) 31](#_Toc1984)

[Table 6.3 Details of SIDE splitting results (24 months and over) 32](#_Toc16607)

[Appendix 7: Sensitivity analyses 34](#_Toc21417)

[Table 7.1 Changes in heterogeneity 34](#_Toc19590)

[Figure 7.1 Exclude studies at overall high risk of bias (6 months) 34](#_Toc19247)

[Figure 7.2 Exclude studies at overall high risk of bias (12 months) 34](#_Toc23716)

[Figure 7.3 Exclude studies at overall high risk of bias (24 months and over) 35](#_Toc25706)

[Appendix 8 Confidence in Network Meta-Analysis (CINeMA) 35](#_Toc11098)

[Figure 8.1 CINeMA netplot (6 months) 35](#_Toc17334)

[Figure 8.2 CINeMA netplot (12 months) 35](#_Toc23372)

[Figure 8.3 CINeMA netplot (24 months and over) 36](#_Toc14842)

[Table 8.1 CINeMA report (6 months) 36](#_Toc5970)

[Table 8.2 CINeMA report (12 months) 39](#_Toc5646)

[Table 8.3 CINeMA report (24 months) 42](#_Toc3261)

[Appendix 9: Additional analyses 44](#_Toc27253)

[Figure 9.1 Network plots for additional analyses comparing (6 months) 44](#_Toc31921)

[Figure 9.2 Network plots for additional analyses comparing (12 months) 44](#_Toc12741)

[Figure 9.3 Network plots for additional analyses comparing (24 months and over) 45](#_Toc17761)

[Figure 9.4 Forest plot for additional analyses (6 months) 45](#_Toc20340)

[Figure 9.5 Forest plot for additional analyses (12 months) 45](#_Toc2617)

[Figure 9.6 Forest plot for additional analyses (24 month and overs) 46](#_Toc6113)

[Table 9.1 SUCRA of prophylactic efficacy on BMD in calcar (additional analyses) 46](#_Toc6706)

[Appendix 10 PRISMA NMA Checklist of Items to Include When Reporting A Systematic Review Involving a Network Meta-analysis 46](#_Toc8659)

# Appendix 1: Search strategy

Table 1.1 Search query and results for each database

| No. | Query | Results |
| --- | --- | --- |
| **Pubmed** | | |
| #1 | (bisphosphonate) or (alendronate) or (zoledronate) or (risedronate) or (ibandronate) or (minodronate) or (pamidronate) or (etidronate) or (clodronate) or (denosumab) or (elcatonin) or (salmon calcitonin) or (MHT) or (SERMs) or (raloxifene) or (bazedoxifene) or (PTHa) or (teriparatide) or (abaloparatide) or (Vitamin D) or (alfacalcidol) or (calcitriol) or (eldecalcitol) or (menatetrenone) or (romosozumab) | 177752 |
| #2 | "Arthroplasty, Replacement, Hip"[Mesh] | 34676 |
| #3 | total and hip and (arthroplasty or replacement) | 45190 |
| #4 | "Arthroplasty, Replacement, Hip"[Mesh] or (total and hip and (arthroplasty or replacement)) | 54680 |
| #5 | ((bisphosphonate) or (alendronate) or (zoledronate) or (risedronate) or (ibandronate) or (minodronate) or (pamidronate) or (etidronate) or (clodronate) or (denosumab) or (elcatonin) or (salmon calcitonin) or (MHT) or (SERMs) or (raloxifene) or (bazedoxifene) or (PTHa) or (teriparatide) or (abaloparatide) or (Vitamin D) or (alfacalcidol) or (calcitriol) or (eldecalcitol) or (menatetrenone) or (romosozumab)) and ("Arthroplasty, Replacement, Hip"[Mesh] or (total and hip and (arthroplasty or replacement))) | 590 |
| **Embase** | | |
| #1 | bisphosphonate OR alendronate OR zoledronate OR risedronate OR ibandronate OR minodronate OR pamidronate OR etidronate OR clodronate OR denosumab OR elcatonin OR (salmon AND calcitonin) OR mht OR serms OR raloxifene OR bazedoxifene OR ptha OR teriparatide OR abaloparatide OR (vitamin AND d) OR alfacalcidol OR calcitriol OR eldecalcitol OR menatetrenone OR romosozumab | 307507 |
| #2 | 'total hip replacement'/exp | 10454 |
| #3 | total AND hip AND (replacement OR arthroplasty) | 59275 |
| #4 | 'total hip replacement'/exp OR (total AND hip AND (replacement OR arthroplasty)) | 59275 |
| #5 | (bisphosphonate OR alendronate OR zoledronate OR risedronate OR ibandronate OR minodronate OR pamidronate OR etidronate OR clodronate OR denosumab OR elcatonin OR (salmon AND calcitonin) OR mht OR serms OR raloxifene OR bazedoxifene OR ptha OR teriparatide OR abaloparatide OR (vitamin AND d) OR alfacalcidol OR calcitriol OR eldecalcitol OR menatetrenone OR romosozumab) AND ('total hip replacement'/exp OR (total AND hip AND (replacement OR arthroplasty))) | 912 |
| **Scopus** | | |
| #1 | ( bisphosphonate ) OR ( alendronate ) OR ( zoledronate ) OR ( risedronate ) OR ( ibandronate ) OR ( minodronate ) OR ( pamidronate ) OR ( etidronate ) OR ( clodronate ) OR ( denosumab ) OR ( elcatonin ) OR ( salmon AND calcitonin ) OR ( mht ) OR ( serms ) OR ( raloxifene ) OR ( bazedoxifene ) OR ( ptha ) OR ( teriparatide ) OR ( abaloparatide ) OR ( vitamin AND d ) OR ( alfacalcidol ) OR ( calcitriol ) OR ( eldecalcitol ) OR ( menatetrenone ) OR ( romosozumab ) | 231548 |
| #2 | Arthroplasty, Replacement, Hip [mesh] | 139 |
| #3 | total AND hip AND ( arthroplasty OR replacement ) | 58838 |
| #4 | Arthroplasty, Replacement, Hip [mesh] OR ( total AND hip AND ( arthroplasty OR replacement ) ) | 23941 |
| #5 | ( ( bisphosphonate ) OR ( alendronate ) OR ( zoledronate ) OR ( risedronate ) OR ( ibandronate ) OR ( minodronate ) OR ( pamidronate ) OR ( etidronate ) OR ( clodronate ) OR ( denosumab ) OR ( elcatonin ) OR ( salmon AND calcitonin ) OR ( mht ) OR ( serms ) OR ( raloxifene ) OR ( bazedoxifene ) OR ( ptha ) OR ( teriparatide ) OR ( abaloparatide ) OR ( vitamin AND d ) OR ( alfacalcidol ) OR ( calcitriol ) OR ( eldecalcitol ) OR ( menatetrenone ) OR ( romosozumab ) ) AND ( "Arthroplasty, Replacement, Hip" [mesh] OR ( total AND hip AND ( arthroplasty OR replacement ) ) ) | 185 |
| **Cochrane library** | | |
| #1 | MeSH descriptor: [Arthroplasty, Replacement, Hip] explode all trees | 2419 |
| #2 | total and hip and (replacement or arthroplasty) | 7053 |
| #3 | #1 or #2 | 7507 |
| #4 | (bisphosphonate) or (Alendronate) or (zoledronic) or (Risedronate) or (Ibandronate) or (Minodronic) or (pamidronate) or (etidronate) or (clodronate) or (denosumab) or (Elcatonin) or (Salmon Calcitonin) or (MHT) or (SERMs) or (raloxifene) or (bazedoxifene) or (PTHa) or (Teriparatide) or (abaloparatide) or (Vitamin D) or (Alfacalcidol) or (calcitriol) or (Eldecalcitol) or (Menatetrenone) or (Romosozumab) | 28694 |
| #5 | #3 and #4 | 315 |
| **Web of science** | | |
| #1 | ((bisphosphonate) or (alendronate) or (zoledronate) or (risedronate) or (ibandronate) or (minodronate) or (pamidronate) or (etidronate) or (clodronate) or (denosumab) or (elcatonin) or (salmon calcitonin) or (MHT) or (SERMs) or (raloxifene) or (bazedoxifene) or (PTHa) or (teriparatide) or (abaloparatide) or (Vitamin D) or (alfacalcidol) or (calcitriol) or (eldecalcitol) or (menatetrenone) or (romosozumab)) | 233360 |
| #2 | (total and hip and (arthroplasty or replacement)) | 66888 |
| #3 | ((bisphosphonate) or (alendronate) or (zoledronate) or (risedronate) or (ibandronate) or (minodronate) or (pamidronate) or (etidronate) or (clodronate) or (denosumab) or (elcatonin) or (salmon calcitonin) or (MHT) or (SERMs) or (raloxifene) or (bazedoxifene) or (PTHa) or (teriparatide) or (abaloparatide) or (Vitamin D) or (alfacalcidol) or (calcitriol) or (eldecalcitol) or (menatetrenone) or (romosozumab)) and (total and hip and (arthroplasty or replacement)) | 846 |

**Appendix 2: Characteristics of studies and subjects included in the NMA**

**Table 2.1 Characteristics of eligible RCTs included in NMA**

| title | authors | year of publication | follow-up time | sample size | mean age | gender distribution(Male/Female) | BMI | medication duration | prosthesis type |
| --- | --- | --- | --- | --- | --- | --- | --- | --- | --- |
| Alendronate prevents femoral periprosthetic bone loss following total hip arthroplasty: prospective randomized doubleblind study | Arabmotlagh M, Rittmeister M, Hennigs T. | 2006 | 12mon | 51 | 62.5 (48–78) 60.8 (47–75) 64.2 (52–72) | 12/12 8/5 6/8 | 28.7 29.1 29.1 | placebo; alendronate 20mg/day 4months; alendronate 20mg/d 2months+alendronate 10mg/d 4months | uncemented |
| Changes of femoral periprosthetic bone mineral density 6 years after treatment with alendronate following total hip arthroplasty | Arabmotlagh M, Pilz M, Warzecha J, Rauschmann M. | 2009 | 6year | 49 | 64 (54–75)  57 (40–68)  58.8 (40–72) | 9/11  9/5  7/8 |  | none; alendronate 10mg/d,10 weeks;  alendronate 20mg/d, 5 weeks | uncemented |
| A longlasting bisphosphonate partially protects periprosthetic bone but does not enhance initial stability of uncemented femoral stems: A randomized placebocontrolled trial of women undergoing total hip arthroplasty | Aro E, Moritz N, Mattila K, Aro HT. | 2018 | 4year | 49 | 71.0 ± 9.5 65.3 ± 8.0 | 0/24 0/25 | 29.8 ± 4.8 28.4 ± 6.5 | The participants were randomized to receive either a single infusion of 5 mg of zoledronic acid or placebo prior to the discharge from the hospital | uncemented |
| Denosumab in cementless total hip arthroplasty: multivariate reanalysis of 3D femoral stem migration and the influence on outliers | Sami Finnilä | 2021 | 48week | 57 | 69.1 ± 6.1 68.6 ± 5.0 | 0/26 0/31 | 28.2 ± 3.8 27.8 ± 5.5 | a clinical dose of 60 mg every 6 months or placebo for 1 year. All subjects received calcium and vitamin D supplements. The first subcutaneous dose of denosumab or placebo was administered 4 weeks before the surgery | uncemented |
| Etidronate does not suppress periprosthetic bone loss following cemented hip arthroplasty | Fokter SK, Komadina R, Repse-Fokter A, Yerby SA, Kocijancic A, Marc J. | 2005 | 12mon | 46 | 70 ± 7 70 ± 6 | 5/15 6/20 | 26.9 ± 3.8 27.4 ± 4.8 | In both groups, the tablets were given in a two-week cycle followed by 12 weeks of calcium supplementation of 260 mg/day. etidronate group received 400 mg/day etidronate during the 14-day cycle. | cemented |
| Effect of etidronate in preventing periprosthetic bone loss following cemented hip arthroplasty: a randomized double blind controlled trial | Fokter SK, Komadina R, Repse-Fokter A. | 2006 | 12mon | 31 | 69 ± 7 68 ± 5 | 3/10 6/12 | 27.7 ± 4.2 28.3 ± 5.0 | In both groups, the tablets were given in a 2-week cycle followed by 12 weeks of calcium supplementation of 260 mg/day . Patients in the etidronate group received 400 mg etidronate/day during the 14-day cycle. | cemented |
| Raloxifene Prevents Early Periprosthetic Bone Loss for Postmenopausal Women after Uncemented Total Hip Arthroplasty: A Randomized Placebo-Controlled Clinical Trial | Gong L, Zhang YY, Yang N, Qian HJ, Zhang LK, Tan MS. | 2020 | 24mon | 240 | 63.2±5.0 62.5±5.4 | 0/120 0/120 | 27.0 ± 3.0 26.8 ± 2.8 | Participants were randomly allocated to receive 60 mg raloxifene hydrochloride per day (treatment group, TG, n = 120) or placebo (control group, CG, n = 120) orally at bedtime | uncemented |
| Bone turnover and periprosthetic bone loss after cementless total hip arthroplasty can be restored by zoledronic acid: a prospective randomized openlabel controlled trial | Huang TW, Wang CJ, Shih HN, Chang Y, Huang KC, Peng KT, Lee MS. | 2017 | 2year | 54 | 59.4 ± 13.3 60.1 ± 11.7 | 14/13 15/12 | 25 (5) 26 (4) | ZA group received 5 mg ZA via intravenous infusion with 0.9% normal saline (500 mL). The control group received only an intravenous saline infusion. All patients received oral calcium (600 mg) and vitamin D3 supplements (800 IU) daily throughout the course of the study | uncemented |
| A comparison of the effects of alendronate and alfacalcidol on bone mineral density around the femoral implant and in the lumbar spine after total hip arthroplasty | Iwamoto N, Inaba Y, Kobayashi N, Ishida T, Yukizawa Y, Saito T. | 2011 | 48week | 54 | 65±10 | 14/46 | 24.3 ± 4.3 | oral administration of alendronate  (5 mg/day) or alfacalcidol (1 jjLg/day) beginning on postoperative Day 1 and  continuing to Week 48. | uncemented |
| The effectiveness of mono or combined osteoporosis drug therapy against bone mineral density loss around femoral implants after total hip arthroplasty | Iwamoto N, Inaba Y, Kobayashi N, Yukizawa Y, Ike H, Ishida T, Saito T. | 2014 | 48week | 60 | 65 ± 9 64 ± 10 65 ± 8 |  | 25.1 ± 3.8 23.6 ± 5.4 24.4 ± 5.1 | Subjects were assigned randomly to the monotherapy group (alendronate 5 mg/day) or non-medication group; combined-therapy group receiving both alendronate (5 mg/day) and alfacalcidol (1 lg/day),The drug treatments were started on postoperative day 1. | uncemented |
| Teriparatide Versus Alendronate for the Preservation of Bone Mineral Density After Total Hip Arthroplasty A randomized Controlled Trial | Kobayashi N, Inaba Y, Uchiyama M, Ike H, Kubota S, Saito T. | 2016 | 12mon | 46 | 65 ± 9 65 ± 10 66 ± 11 | 1/15 1/13  4/12 | 24.6 ± 4.0 23.8 ± 2.8  21.9 ± 4.3 | Drug administrations were performed by subcutaneous daily injection of teriparatide (Forteo 20 μg/day), or oral weekly administration of alendronate (Bonalon 35 mg/week), beginning on postoperative week 2 and continuing until week 48. | uncemented |
| Effect of switching administration of alendronate after teriparatide for the prevention of BMD loss around the implant after total hip arthroplasty 2year followup: a randomized controlled trial | Morita A, Kobayashi N, Choe H, Ike H, Tezuka T, Higashihira S, Inaba Y. | 2020 | 2year | 47 | 64.8 ± 9.2 63.8 ± 9.3  65.8 ± 10.6 | 4/11 1/14 1/16 | 24.6 ± 3.8 24.0 ± 2.6  21.9 ± 4.2 | switch group were started on daily injections of 20 μg/day teriparatide, beginning 2 weeks after THA and continuing for 1 year switched to oral administration of ALD (35mg/week) continuously for 1 year. ALD group were started on oral administration of alendronate (35 mg/week) beginning 2 weeks after THA and continuing for 2 years. Patients in the control group received no drug for 2 years. | uncemented |
| Restoration of proximal periprosthetic bone loss by denosumab in cementless total hip arthroplasty | Nagoya S, Tateda K, Okazaki S, Kosukegawa I, Shimizu J, Yamashita T. | 2018 | 1year | 20 | 80.8 ± 2.2 78.4 ± 4.3 | 0/10 0/10 |  | The denosumab group was administered  60 mg denosumab subcutaneously from 1 week to 2 weeks after THA and received a subcutaneous administration again at 6 months after surgery. an active form of vitamin D (eldecalcitol; 0.75 μg) was given by oral administration every day to prevent hypocalcemia. | uncemented |
| Denosumab prevented periprosthetic bone resorption better than risedronate after total hip arthroplasty | Nakura N, Hirakawa K, Takayanagi S, Mihara M. | 2023 | 24mon | 82 | 69.9 ± 5.00  68.3 ± 6.32 | 1/39  4/38 |  | The denosumab group received 60 mg of subcutaneous denosumab on postoperative day 1 and every 6 months for 2 years; the risedronate group received 17.5 mg of oral risedronate on postoperative day 3 and weekly thereafter. Throughout the study, the denosumab group received daily  supplements of 600 mg of calcium and 400 IU of vitamin D to prevent hypocalcemia | uncemented |
| Alendronate inhibits periprosthetic bone loss around uncemented femoral components | Takashi Nishioka, et al. | 2007 | 12mon | 15 | 63.2±3.4 63.4±1.3 | 1/8 0/8 |  | 5 mg/day oral alendronate immediately after arising in the morning for 1 year | uncemented |
| Effect of zoledronic acid on reducing femoral bone mineral density loss following total hip arthroplasty: preliminary results of a prospective randomized trial | Scott DF, Woltz JN, Smith RR. | 2013 | 2year | 51 | 62.9 (51–72)/60.7 (51–77) 55.8 (33–76)/ 64.7 (52–84) | 11/13 12/15 | 30.0 (25.1–33.5)/29.4 (21.3–37.6) 28.3 (22.4–36.7)/ 29.8 (22.3–40.2) | intravenous infusion of ZOL 5mg or saline at 14days and at 1 year after primary cementless THA. all participants took 1500mg of calcium carbonate and 600 IU of vitamin D daily for the 2-year study | uncemented |
| The effect of weekly risedronate on periprosthetic bone resorption following total hip arthroplasty: a randomized doubleblind placebocontrolled trial | Sköldenberg OG, Salemyr MO, Bodén HS, Ahl TE, Adolphson PY. | 2011 | 2year | 73 | 60 ± 7 61 ± 7 | 21/16 22/14 | 28 ± 6 27 ± 4 | 35 mg of risedronate or the placebo once weekly for six months. All patients also received calcium carbonate (1000 mg) and vitamin D(400 IU) oral supplements daily for six months. | uncemented |
| Alendronate reduces periprosthetic bone loss after uncemented primary total hip arthroplasty a 5year followup of 16 patients | Tapaninen TS, Venesmaa PK, Jurvelin JS, Miettinen HJ, Kröger HP. | 2010 | 5year | 16 | 59 (46–70) 65 (59–71) | 5/4  2/5 | 27.8 (22.3–44.3)  28.5 (23.0–36.2) | 500 mg calcium carbonate daily.  10 mg alendronate sodium supplemented by 500 mg calcium carbonate daily. Alendronate tablets were administrated orally 30 minutes before the breakfast, begin_x005f_x005f_x005f_x005f_x005f_x005f_x005f_x005f_x005f_x005f_x005f_x005f_x005f_x005f_x005f_x0002_ning the first day after surgery. The duration of the treat ment was 6 months. | uncemented |
| Decreased periprosthetic bone loss in patients treated with clodronate: a 1year randomized controlled study | Trevisan C, Ortolani S, Romano P, Isaia G, Agnese L, Dallari D, Grappiolo G, Cherubini R, Massari L, Bianchi G. | 2010 | 12mon | 91 | 65.6 ± 6.4 63.7 ± 7.8 | 27/22 26/16 | 27.2 (3.9) 26.8 (3.3) | Patients assigned to active treatment received 100 mg/ day of clodronate intramuscularly for 10 consecutive days and thereafter weekly for 50 weeks | uncemented |
| Alendronate Reduces Periprosthetic Bone Loss After Uncemented Primary Total Hip Arthroplasty: A Prospective Randomized Study | PETRI K. VENESMAA | 2001 | 6mon | 13 | 62 (57–70)  63 (50–71) | 3/2 3/5 | 24.9 (23.3–29.8)  28.3 (23.0–36.2) | 500 mg calcium carbonate daily; 10 mg alendronate sodium. 500 mg calcium carbonate daily. Supplements were ad_x005f ministered orally 30 minutes before breakfast, beginning on the first day after surgery. | uncemented |
| Cyclic therapy with etidronate has a therapeutic effect against local osteoporosis after cementless total hip arthroplasty | Yamaguchi K, Masuhara K, Yamasaki S, Nakai T, Fuji T. | 2003 | 12mon | 52 | 67 ± 6 70 ± 9 | 5/25 6/16 |  | Group A without osteoactive drugs. Group B with cyclic therapy with etidronate (i.e., 400 mg/day of oral etidronate for 2 weeks followed by 12 weeks of 500 mg/day of calcium lactate and repeated every 14 weeks) | uncemented |
| Effects of discontinuation as well as intervention of cyclic therapy with etidronate on bone remodeling after cementless total hip arthroplasty | Yamaguchi K, Masuhara K, Yamasaki S, Fuji T, Seino Y. | 2004 | 30mon | 55 | 64 ± 8 70 ± 6 65 ± 5 | 2/22 4/12 1/14 |  | CTE12-18mon;Sixteen patients continued on CTE (i.e., 400 mg/day oral etidronate for 2 weeks followed by 12 weeks of 500 mg/day calcium lactate, repeated every 14 weeks) for the first 12 months followed by no treatment for 18 months (early-etidronate group). Fifteen patients received no treatment for the first 18 months followed by CTE for 12 months (lateetidronate group). | uncemented |
| Efficacy of different dosing schedules of etidronate for stress shielding after cementless total hip arthroplasty | Yamaguchi K, Masuhara K, Yamasaki S, Fuji T. | 2005 | 12mon | 43 | 68 ± 3 66 ± 6 70 ± 10 | 0/17 0/12 0/14 |  | two groups received 200mg or 400 mg etidronate disodium once a day in a 2-week cycle followed by 12 weeks of calcium lactate of 500mg/day. This cycle was repeated four times, starting on the 7th day after surgery. | uncemented |
| Risedronate reduces postoperative bone resorption after cementless total hip arthroplasty | Yamasaki S, Masuhara K, Yamaguchi K, Nakai T, Fuji T, Seino Y. | 2007 | 6mon | 40 | 66.7 ± 4.4  66.8 ± 6.5+ | 2/19  2/17 |  | no osteoactive drug(calcium lactate 3 g/day) or oral risedronate 2.5 mg/day for 6 months | uncemented |
| Efficacy of Alendronate for the Prevention of Bone Loss in Calcar Region Following Total Hip Arthroplasty | Yukizawa Y, Inaba Y, Kobayashi N, Choe H, Kubota S, Saito T. | 2017 | 9year | 60 | 64 ± 10 63 ± 10 64 ± 7 | 7/9 4/14 2/10 |  | alendronate (5 mg/day) in the alendronate group and alfacalcidol (1 µg/day) in the alfacalcidol group were started on the day after surgery and continued for at least 2 years. | uncemented |
| Effects of zoledronic acid on bone mineral density around prostheses and bone metabolism markers after primary total hip arthroplasty in females with postmenopausal osteoporosis | Zhou W, Liu Y, Guo X, Yang H, Xu Y, Geng D. | 2019 | 12mon | 32 | 74.4 ± 5.7 73.3 ± 6.6 | 0/16 0/16 | 24.4 ± 4.2 23.9 ± 3.5 | control group: oral calcium carbonate 1200 mg/day and calcitriol 0.50 μg/ day; ZOL group: added an intravenous infusion of 5 mg zoledronic acid at 5–7 days after operation on the basis of the basic treatment | uncemented |

**Table 2.2 Data in NMA (6months)**

|  | n1 | mean1 | sd1 | n2 | mean2 | sd2 | n3 | mean3 | sd3 | t1 | t2 | t3 |
| --- | --- | --- | --- | --- | --- | --- | --- | --- | --- | --- | --- | --- |
| Arabmotlagh2006 | 24 | -0.23835345 | 0.189675525 | 13 | -0.0767071 | 0.100358774 | 14 | -0.0889286 | 0.191701412 | Placebo | Alendronate | Alendronate |
| Aro2018 | 24 | -0.10389715 | 0.146547187 | 25 | 0.0376672 | 0.230005005 |  |  |  | Placebo | Zoledronate |  |
| Finnilä2021 | 26 | -0.1654 | 0.0978 | 31 | -0.0698 | 0.0942 |  |  |  | Placebo | Denosumab |  |
| Fokter2005 | 20 | -0.141 | 0.178 | 26 | -0.105 | 0.241 |  |  |  | Placebo | Etidronate |  |
| Fokter2006 | 13 | -0.234 | 0.112 | 18 | -0.129 | 0.268 |  |  |  | Placebo | Etidronate |  |
| Gong2020 | 120 | -0.09253 | 0.06825 | 120 | -0.07656 | 0.05521 |  |  |  | Placebo | Raloxifene |  |
| Huang2017 | 27 | -0.12 | 0.29 | 27 | 0.09 | 0.2 |  |  |  | Placebo | Zoledronate |  |
| Iwamoto2011 | 22 | -0.206 | 0.211 | 18 | -0.081 | 0.154 | 14 | -0.27 | 0.11 | Placebo | Alendronate | Alfacalcidol |
| Iwamoto2014 | 22 | -0.22 | 0.18 | 18 | -0.08 | 0.15 | 20 | -0.08 | 0.13 | Placebo | Alendronate | Alendronate+Alfacalcidol |
| Morita2020 | 16 | -0.247 | 0.148 | 15 | -0.096 | 0.081 | 17 | -0.123 | 0.065 | Placebo | Alendronate | Teriparatide |
| Nagoya2018 | 10 | -0.16446 | 0.291742483 | 10 | 0.02712 | 0.278322608 |  |  |  | Placebo | Denosumab |  |
| Nakura2023 | 40 | 0.029 | 0.073 | 42 | -0.131 | 0.097 |  |  |  | Denosumab | Risedronate |  |
| Nishioka2007 | 8 | -0.182 | 0.082 | 7 | -0.037 | 0.112 |  |  |  | Placebo | Alendronate |  |
| Scott2013 | 24 | -0.228 | 0.115 | 27 | -0.0571 | 0.098 |  |  |  | Placebo | Zoledronate |  |
| Sköldenberg2011 | 37 | -0.128 | 0.06 | 36 | -0.05 | 0.05 |  |  |  | Placebo | Risedronate |  |
| Tapaninen2010 | 9 | -0.1426 | 0.05 | 7 | 0.0014 | 0.092 |  |  |  | Placebo | Alendronate |  |
| Trevisan2010 | 42 | -0.222 | 0.022 | 38 | -0.215 | 0.024 |  |  |  | Placebo | Clodronate |  |
| Venesmaa2001 | 5 | -0.177 | 0.082 | 8 | -0.009 | 0.159 |  |  |  | Placebo | Zoledronate |  |
| Yamaguchi2003 | 30 | -0.241 | 0.12 | 22 | -0.141 | 0.125 |  |  |  | Placebo | Etidronate |  |
| Yamaguchi2004 | 24 | -0.209 | 0.148 | 16 | -0.141 | 0.125 | 15 | -0.279 | 0.093 | Placebo | Etidronate | Etidronate |
| Yamaguchi2005 | 17 | -0.251 | 0.129 | 12 | -0.182 | 0.103 | 14 | -0.149 | 0.114 | Placebo | Etidronate | Etidronate |
| Yamasaki2007 | 21 | -0.202 | 0.103 | 19 | -0.119 | 0.1 |  |  |  | Placebo | Risedronate |  |
| Zhou2019 | 16 | -0.227 | 0.059 | 16 | -0.16 | 0.062 |  |  |  | Placebo | Zoledronate |  |

**Table 2.3 Data in NMA (12months)**

|  | n1 | mean1 | sd1 | n2 | mean2 | sd2 | n3 | mean3 | sd3 | t1 | t2 | t3 |
| --- | --- | --- | --- | --- | --- | --- | --- | --- | --- | --- | --- | --- |
| Arabmotlagh2006 | 24 | -0.26 | 0.213157457 | 13 | -0.224 | 0.181638008 | 14 | -0.102 | 0.173729367 | Placebo | Alendronate | Alendronate |
| Arabmotlagh2009 | 20 | -0.286 | 0.134 | 14 | -0.228 | 0.094 | 15 | -0.209 | 0.136 | Placebo | Alendronate | Alendronate |
| Aro2018 | 24 | -0.05769805 | 0.157588411 | 25 | 0.03321685 | 0.274671081 |  |  |  | Placebo | Zoledronate |  |
| Finnilä2021 | 26 | -0.1682 | 0.1015 | 31 | -0.0471 | 0.0881 |  |  |  | Placebo | Denosumab |  |
| Fokter2005 | 20 | -0.166 | 0.188 | 26 | -0.058 | 0.333 |  |  |  | Placebo | Etidronate |  |
| Fokter2006 | 13 | -0.255 | 0.117 | 18 | -0.113 | 0.368 |  |  |  | Placebo | Etidronate |  |
| Gong2020 | 120 | -0.15725 | 0.06877 | 120 | -0.08878 | 0.07565 |  |  |  | Placebo | Raloxifene |  |
| Huang2017 | 27 | -0.16 | 0.29 | 27 | 0.07 | 0.22 |  |  |  | Placebo | Zoledronate |  |
| Iwamoto2011 | 22 | -0.27 | 0.16 | 18 | -0.08 | 0.22 | 14 | -0.27 | 0.13 | Placebo | Alendronate | Alfacalcidol |
| Iwamoto2014 | 22 | -0.27 | 0.16 | 18 | -0.08 | 0.22 | 20 | -0.09 | 0.17 | Placebo | Alendronate | Alendronate_Alfacalcidol |
| Kobayashi2016 | 16 | -0.362 | 0.133 | 14 | -0.161 | 0.185 | 16 | -0.106 | 0.162 | Placebo | Alendronate | Teriparatide |
| Morita2020 | 15 | -0.3198488 | 0.143693305 | 15 | -0.1683849 | 0.070031948 | 16 | -0.1219171 | 0.06634621 | Placebo | Alendronate | Teriparatide+Alendronate |
| Nagoya2018 | 10 | -0.23041 | 0.318582232 | 10 | 0.07262 | 0.29990624 |  |  |  | Placebo | Denosumab |  |
| Nakura2023 | 40 | 0.055 | 0.091 | 42 | -0.212 | 0.115 |  |  |  | Denosumab | Risedronate |  |
| Nishioka2007 | 8 | -0.196 | 0.187 | 7 | -0.045 | 0.109 |  |  |  | Placebo | Alendronate |  |
| Scott2013 | 24 | -0.254 | 0.109 | 27 | -0.0839 | 0.125 |  |  |  | Placebo | Zoledronate |  |
| Sköldenberg2011 | 37 | -0.16 | 0.12 | 36 | -0.12 | 0.061 |  |  |  | Placebo | Risedronate |  |
| Tapaninen2010 | 9 | -0.201 | 0.087 | 7 | -0.037 | 0.095 |  |  |  | Placebo | Alendronate |  |
| Trevisan2010 | 32 | -0.288 | 0.03 | 28 | -0.223 | 0.028 |  |  |  | Placebo | Clodronate |  |
| Yamaguchi2003 | 30 | -0.302 | 0.117 | 22 | -0.14 | 0.124 |  |  |  | Placebo | Etidronate |  |
| Yamaguchi2004 | 24 | -0.291 | 0.136 | 16 | -0.141 | 0.133 | 15 | -0.308 | 0.118 | Placebo | Etidronate | Etidronate |
| Yamaguchi2005 | 17 | -0.301 | 0.112 | 12 | -0.159 | 0.149 | 14 | -0.156 | 0.116 | Placebo | Etidronate | Etidronate |
| Yukizawa2017 | 16 | -0.27 | 0.16 | 18 | -0.08 | 0.22 | 12 | -0.24 | 0.13 | Placebo | Alendronate | Alfacalcidol |
| Zhou2019 | 16 | -0.277 | 0.06 | 16 | -0.197 | 0.082 |  |  |  | Placebo | Zoledronate |  |

**Table 2.4 Data in NMA (24months and over)**

|  | n1 | mean1 | sd1 | n2 | mean2 | sd2 | n3 | mean3 | sd3 | t1 | t2 | t3 |
| --- | --- | --- | --- | --- | --- | --- | --- | --- | --- | --- | --- | --- |
| Arabmotlagh2009 | 20 | -0.315 | 0.145 | 14 | -0.226 | 0.109 | 15 | -0.216 | 0.129 | Placebo | Alendronate | Alendronate |
| Aro2018 | 24 | -0.2795811 | 0.201251369 | 25 | -0.1375929 | 0.17609756 |  |  |  | Placebo | Zoledronate |  |
| Gong2020 | 120 | -0.23665 | 0.0712 | 120 | -0.09885 | 0.08 |  |  |  | Placebo | Raloxifene |  |
| Huang2017 | 27 | -0.17 | 0.2 | 27 | 0.06 | 0.22 |  |  |  | Placebo | Zoledronate |  |
| Morita2020 | 15 | -0.354 | 0.107 | 12 | -0.182 | 0.225 | 14 | -0.157 | 0.156 | Placebo | Alendronate | Teriparatide+Alendronate |
| Nakura2023 | 40 | 0.059 | 0.116 | 42 | -0.192 | 0.119 |  |  |  | Denosumab | Risedronate |  |
| Scott2013 | 24 | -0.273 | 0.114 | 27 | -0.0958 | 0.124 |  |  |  | Placebo | Zoledronate |  |
| Sköldenberg2011 | 37 | -0.18 | 0.15 | 36 | -0.17 | 0.069 |  |  |  | Placebo | Risedronate |  |
| Tapaninen2010 | 9 | -0.231 | 0.146 | 7 | -0.1355 | 0.19 |  |  |  | Placebo | Alendronate |  |
| Yamaguchi2004 | 24 | -0.277 | 0.113 | 16 | -0.193 | 0.116 | 15 | -0.294 | 0.131 | Placebo | Etidronate | Etidronate |
| Yukizawa2017 | 16 | -0.41 | 0.17 | 18 | -0.24 | 0.27 | 12 | -0.36 | 0.15 | Placebo | Alendronate | Alfacalcidol |

## List of included studies

Arabmotlagh, Mohammad, Mathias Pilz, Jörg Warzecha, and Michael Rauschmann. 2009. “Changes of Femoral Periprosthetic Bone Mineral Density 6 Years after Treatment with Alendronate Following Total Hip Arthroplasty.” *Journal of Orthopaedic Research : Official Publication of the Orthopaedic Research Society* 27 (2): 183–88. <https://doi.org/10.1002/jor.20748>.

Arabmotlagh, Mohammad, Markus Rittmeister, and Thorsten Hennigs. 2006. “Alendronate Prevents Femoral Periprosthetic Bone Loss Following Total Hip Arthroplasty: Prospective Randomized Double-Blind Study.” *Journal of Orthopaedic Research : Official Publication of the Orthopaedic Research Society* 24 (7): 1336–41. <https://doi.org/10.1002/jor.20162>.

Aro, Erik, Niko Moritz, Kimmo Mattila, and Hannu T. Aro. 2018. “A Long-Lasting Bisphosphonate Partially Protects Periprosthetic Bone, but Does Not Enhance Initial Stability of Uncemented Femoral Stems: A Randomized  Placebo-Controlled Trial of Women Undergoing Total Hip Arthroplasty.” *Journal of Biomechanics* 75 ((Aro E., erik.aro@utu.fi; Moritz N., niko.moritz@utu.fi; Aro H.T., hannu.aro@utu.fi) Department of Orthopedic Surgery and Traumatology, Turku University Hospital and University of Turku, Turku, Finland): 35–45. <https://doi.org/10.1016/j.jbiomech.2018.04.041>.

Aro, Hannu T., Sanaz Nazari-Farsani, Mia Vuopio, Eliisa Löyttyniemi, and Kimmo Mattila. 2019. “Effect of Denosumab on Femoral Periprosthetic BMD and Early Femoral Stem Subsidence in Postmenopausal Women Undergoing uncemented Total Hip Arthroplasty: EFFECT OF DENOSUMAB ON FEMORAL PERIPROSTHETIC BMD.” *JBMR Plus* 3 (10): e10217–e10217. <https://doi.org/10.1002/jbm4.10217>.

Finnilä, Sami, Eliisa Löyttyniemi, and Hannu T Aro. 2022. “Denosumab in uncemented Total Hip Arthroplasty: Multivariate Reanalysis of 3D Femoral Stem Migration and the Influence on Outliers.” *JBMR Plus* 6 (2): e10588–e10588. <https://doi.org/10.1002/jbm4.10588>.

Fokter, S. K., R. Komadina, A. Repše-Fokter, S. A. Yerby, A. Kocijančič, and J. Marc. 2005. “Etidronate Does Not Suppress Periprosthetic Bone Loss Following Cemented Hip Arthroplasty.” *International Orthopaedics* 29 (6): 362–67. <https://doi.org/10.1007/s00264-005-0018-2>.

Fokter, Samo K., Radko Komadina, and Alenka Repše-Fokter. 2006. “Effect of Etidronate in Preventing Periprosthetic Bone Loss Following Cemented Hip Arthroplasty: A Randomized, Double Blind, Controlled Trial.” *Wiener Klinische Wochenschrift, Supplement* 118 Suppl 2 ((Fokter S.K., samo.fokter@guest.arnes.si) Department of Orthopedic Surgery and Sports Trauma, Celje General Hospital, Celje, Slovenia): 23–28. <https://doi.org/10.1007/s00508-006-0556-7>.

Gong, Long, Yao‐yao Zhang, Na Yang, Huan‐juan Qian, Ling‐kun Zhang, and Ming‐sheng Tan. 2020. “Raloxifene Prevents Early Periprosthetic Bone Loss for Postmenopausal Women after Uncemented Total Hip Arthroplasty: A Randomized Placebo-Controlled Clinical  Trial.” *Orthopaedic Surgery* 12 (4): 1074–83. <https://doi.org/10.1111/os.12696>.

Hsu, Allen Herng Shouh, Chun-Hsien Yen, Feng-Chih Kuo, Cheng-Ta Wu, Tsan-Wen Huang, Juei-Tang Cheng, and Mel S. Lee. 2022. “Zoledronic Acid Ameliorates the Bone Turnover Activity and Periprosthetic Bone Preservation in uncemented Total Hip Arthroplasty.” *Pharmaceuticals (Basel, Switzerland)* 15 (4): 420. <https://doi.org/10.3390/ph15040420>.

Huang, Tsan-Wen, Chao-Jan Wang, Hsin-Nung Shih, Yuhan Chang, Kuo-Chin Huang, Kuo-Ti Peng, and Mel S. Lee. 2017. “Bone Turnover and Periprosthetic Bone Loss after uncemented Total Hip Arthroplasty Can Be Restored by Zoledronic Acid: A Prospective, Randomized,  Open-Label, Controlled Trial.” *BMC Musculoskeletal Disorders* 18 (1): 209. <https://doi.org/10.1186/s12891-017-1577-2>.

Iwamoto, Naoyuki, Yutaka Inaba, Naomi Kobayashi, Takashi Ishida, Yohei Yukizawa, and Tomoyuki Saito. 2011. “A Comparison of the Effects of Alendronate and Alfacalcidol on Bone Mineral Density around the Femoral Implant and in the Lumbar Spine after Total Hip  Arthroplasty.” *The Journal of Bone and Joint Surgery. American Volume* 93A (13): 1203–9. <https://doi.org/10.2106/JBJS.I.01714>.

Iwamoto, Naoyuki, Yutaka Inaba, Naomi Kobayashi, Yohei Yukizawa, Hiroyuki Ike, Takashi Ishida, and Tomoyuki Saito. 2014. “The Effectiveness of Mono or Combined Osteoporosis Drug Therapy against Bone Mineral Density Loss around Femoral Implants after Total Hip Arthroplasty.” *Journal of Bone and Mineral Metabolism* 32 (5): 539–44. <https://doi.org/10.1007/s00774-013-0526-x>.

Kobayashi, Naomi, Yutaka Inaba, Makoto Uchiyama, Hiroyuki Ike, So Kubota, and Tomoyuki Saito. 2016. “Teriparatide Versus Alendronate for the Preservation of Bone Mineral Density After Total Hip Arthroplasty - A Randomized Controlled Trial.” *The Journal of Arthroplasty* 31 (1): 333–38. <https://doi.org/10.1016/j.arth.2015.07.017>.

Morita, Akira, Naomi Kobayashi, Hyonmin Choe, Hiroyuki Ike, Taro Tezuka, Shota Higashihira, and Yutaka Inaba. 2020. “Effect of Switching Administration of Alendronate after Teriparatide for the Prevention of BMD Loss around the Implant after Total Hip Arthroplasty, 2-Year  Follow-up: A Randomized Controlled Trial.” *Journal of Orthopaedic Surgery and Research* 15 (1): 17. <https://doi.org/10.1186/s13018-020-1547-5>.

Muren, Olle, Ehsan Akbarian, Mats Salemyr, Henrik Bodén, Thomas Eisler, André Stark, and Olof Sköldenberg. 2015. “No Effect of Risedronate on Femoral Periprosthetic Bone Loss Following Total Hip Arthroplasty. A 4-Year Follow-up of 61 Patients in a Double-Blind, Randomized  Placebo-Controlled Trial.” *Acta Orthopaedica* 86 (5): 569–74. <https://doi.org/10.3109/17453674.2015.1041846>.

Nagoya, Satoshi, Kenji Tateda, Shunichiro Okazaki, Ima Kosukegawa, Junya Shimizu, and Toshihiko Yamashita. 2018. “Restoration of Proximal Periprosthetic Bone Loss by Denosumab in uncemented Total Hip Arthroplasty.” *European Journal of Orthopaedic Surgery & Traumatology : Orthopedie Traumatologie* 28 (8): 1601–7. <https://doi.org/10.1007/s00590-018-2223-x>.

Nakura, Nariaki, Kazuo Hirakawa, Satoshi Takayanagi, and Masahiko Mihara. 2023. “Denosumab Prevented Periprosthetic Bone Resorption Better than Risedronate after Total Hip Arthroplasty.” *Journal of Bone and Mineral Metabolism* 41 (2): 239–47. <https://doi.org/10.1007/s00774-023-01405-2>.

Nishioka, Takashi, Shoji Yagi, Tadashi Mitsuhashi, Masafumi Miyamoto, Tatsuya Tamura, Toru Kobayashi, and Tetsuya Enishi. 2007. “Alendronate Inhibits Periprosthetic Bone Loss around Uncemented Femoral Components.” *Journal of Bone and Mineral Metabolism* 25 (3): 179–83. <https://doi.org/10.1007/s00774-006-0743-7>.

Nyström A, Kiritopoulos D, Ullmark G, Sörensen J, Petrén-Mallmin M, Milbrink J, Hailer NP, Mallmin H. Denosumab Prevents Early Periprosthetic Bone Loss After Uncemented Total Hip Arthroplasty: Results from a Randomized Placebo-Controlled Clinical Trial. J Bone Miner Res. 2020 Feb;35(2):239-247. doi: 10.1002/jbmr.3883. Epub 2019 Nov 4. PMID: 31589776.

Scott, David F., Jennifer N. Woltz, and Rachel R. Smith. 2013. “Effect of Zoledronic Acid on Reducing Femoral Bone Mineral Density Loss Following Total Hip Arthroplasty: Preliminary Results of a Prospective Randomized Trial.” *The Journal of Arthroplasty* 28 (4): 671–75. <https://doi.org/10.1016/j.arth.2012.08.007>.

Sköldenberg, Olof Gustaf, Mats Olof Salemyr, Henrik Stefan Bodén, Torbjörn Efraim Ahl, and Per Yngve Adolphson. 2011. “The Effect of Weekly Risedronate on Periprosthetic Bone Resorption Following Total Hip Arthroplasty: A Randomized, Double-Blind, Placebo-Controlled Trial.” *The Journal of Bone and Joint Surgery. American Volume* 93A (20): 1857–64. <https://doi.org/10.3928/01477447-20111021-23>.

Tapaninen, T. S., P. K. Venesmaa, J. S. Jurvelin, H. J. A. Miettinen, and H.P.J. Kröger. 2010. “Alendronate Reduces Periprosthetic Bone Loss after Uncemented Primary Total Hip Arthroplasty - a 5-Year Follow-up of 16 Patients.” *Scandinavian Journal of Surgery : SJS : Official Organ for the Finnish Surgical Society and the Scandinavian Surgical Society* 99 (1): 32–37. <https://doi.org/10.1177/145749691009900108>.

Trevisan, C., S. Ortolani, P. Romano, G. Isaia, L. Agnese, D. Dallari, G. Grappiolo, R. Cherubini, L. Massari, and G. Bianchi. 2010. “Decreased Periprosthetic Bone Loss in Patients Treated with Clodronate: A 1-Year Randomized Controlled Study.” *Calcified Tissue International* 86 (6): 436–46. <https://doi.org/10.1007/s00223-010-9356-1>.

Venesmaa, Petri K., Heikki P. J. Kröger, Hannu J. A. Miettinen, Jukka S. Jurvelin, Olavi T. Suomalainen, and Esko M. Alhava. 2001. “Alendronate Reduces Periprosthetic Bone Loss after Uncemented Primary Total Hip Arthroplasty: A Prospective Randomized Study.” *Journal of Bone and Mineral Research : The Official Journal of the American Society for Bone and Mineral Research* 16 (11): 2126–31. <https://doi.org/10.1359/jbmr.2001.16.11.2126>.

Yamaguchi, Katsuyuki, Kensaku Masuhara, Satoshi Yamasaki, and Takeshi Fuji. 2005. “Efficacy of Different Dosing Schedules of Etidronate for Stress Shielding after uncemented Total Hip Arthroplasty.” *Journal of Orthopaedic Science : Official Journal of the Japanese Orthopaedic Association* 10 (1): 32–36. <https://doi.org/10.1007/s00776-004-0854-8>.

Yamaguchi, Katsuyuki, Kensaku Masuhara, Satoshi Yamasaki, Takeshi Fuji, and Yoshiki Seino. 2004. “Effects of Discontinuation as Well as Intervention of Cyclic Therapy with Etidronate on Bone Remodeling after uncemented Total Hip Arthroplasty.” *Bone* 35 (1): 217–23. <https://doi.org/10.1016/j.bone.2004.03.017>.

Yamaguchi, Katsuyuki, Kensaku Masuhara, Satoshi Yamasaki, Tsuyoshi Nakai, and Takeshi Fuji. 2003. “Cyclic Therapy with Etidronate Has a Therapeutic Effect against Local Osteoporosis after uncemented Total Hip Arthroplasty.” *Bone* 33 (1): 144–49. <https://doi.org/10.1016/S8756-3282(03)00085-1>.

Yamasaki, S., K. Masuhara, K. Yamaguchi, T. Nakai, T. Fuji, and Y. Seino. 2007. “Risedronate Reduces Postoperative Bone Resorption after uncemented Total Hip Arthroplasty.” *Osteoporosis International : A Journal Established as Result of Cooperation between the European Foundation for Osteoporosis and the National Osteoporosis  Foundation of the USA* 18 (7): 1009–15. <https://doi.org/10.1007/s00198-007-0339-7>.

Yukizawa, Yohei, Yutaka Inaba, Naomi Kobayashi, Hyonmin Choe, So Kubota, and Tomoyuki Saito. 2017. “Efficacy of Alendronate for the Prevention of Bone Loss in Calcar Region Following Total Hip Arthroplasty.” *The Journal of Arthroplasty* 32 (7): 2176–80. <https://doi.org/10.1016/j.arth.2017.02.036>.

Zhou, W., Y. Liu, X. Guo, H. Yang, Y. Xu, and D. Geng. 2019. “Effects of Zoledronic Acid on Bone Mineral Density around Prostheses and Bone Metabolism Markers after Primary Total Hip Arthroplasty in Females with  Postmenopausal Osteoporosis.” *Osteoporosis International : A Journal Established as Result of Cooperation between the European Foundation for Osteoporosis and the National Osteoporosis  Foundation of the USA* 30 (8): 1581–89. <https://doi.org/10.1007/s00198-019-05005-7>.

# Appendix 3: The risk of bias assessment for the individual included studies.

**Figure 3.1 ROB2 figure (ITT)**


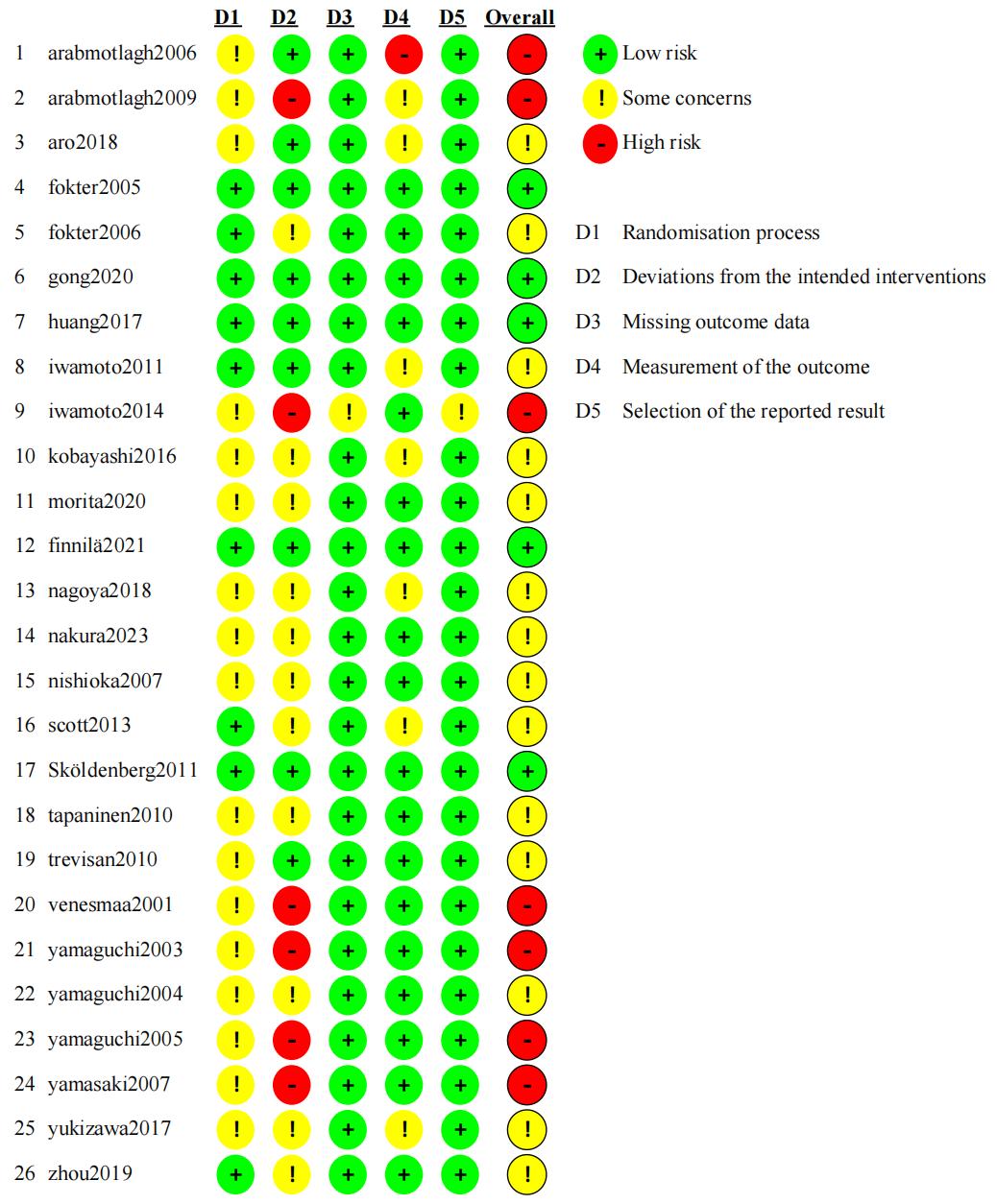


# Appendix 4: Diagnosis of convergence

## Figure 4.1 Gelman plot (6 months)


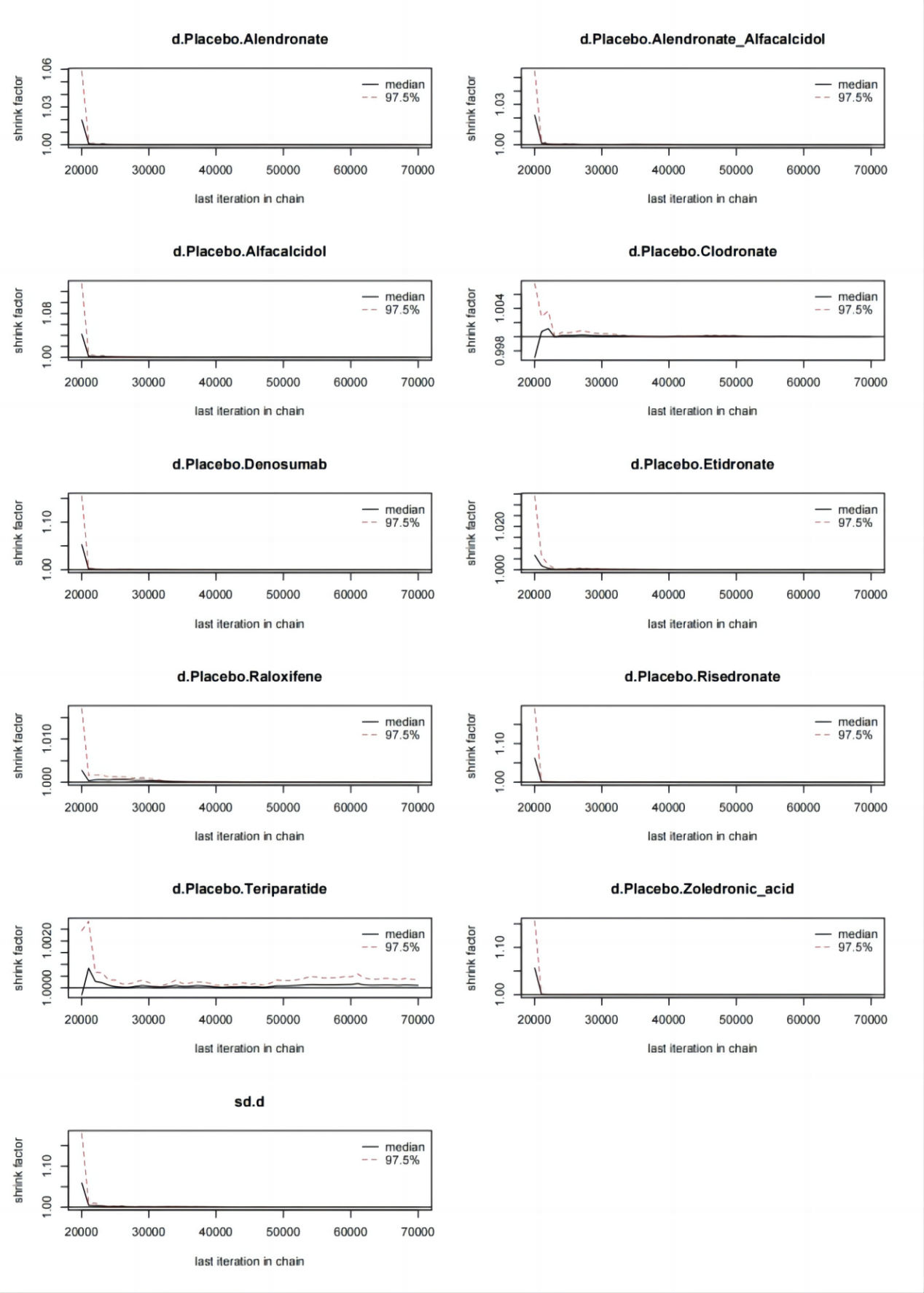


## Figure 4.2 Gelman plot (12 months)


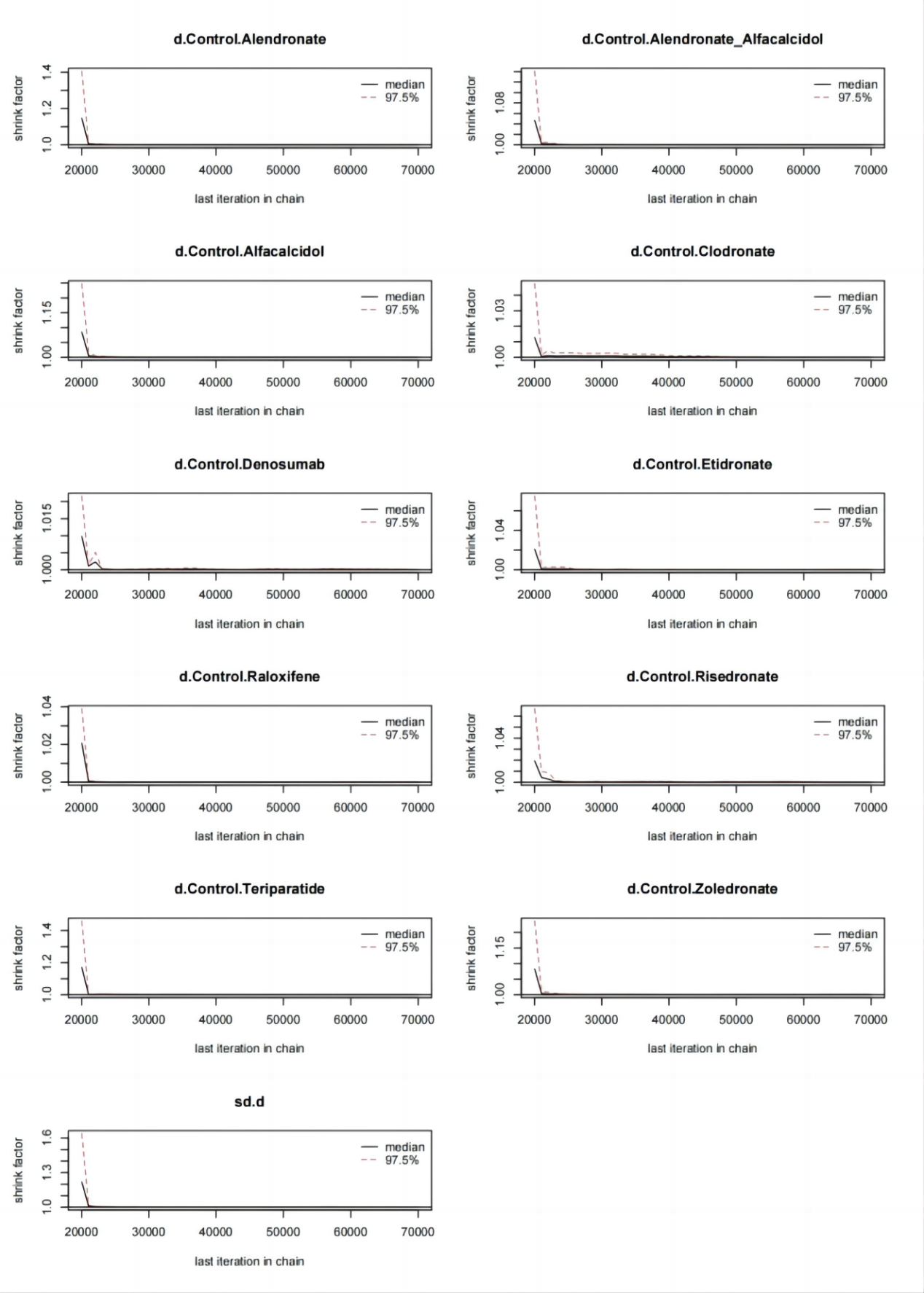


## Figure 4.3 Gelman plot (24 months and over)


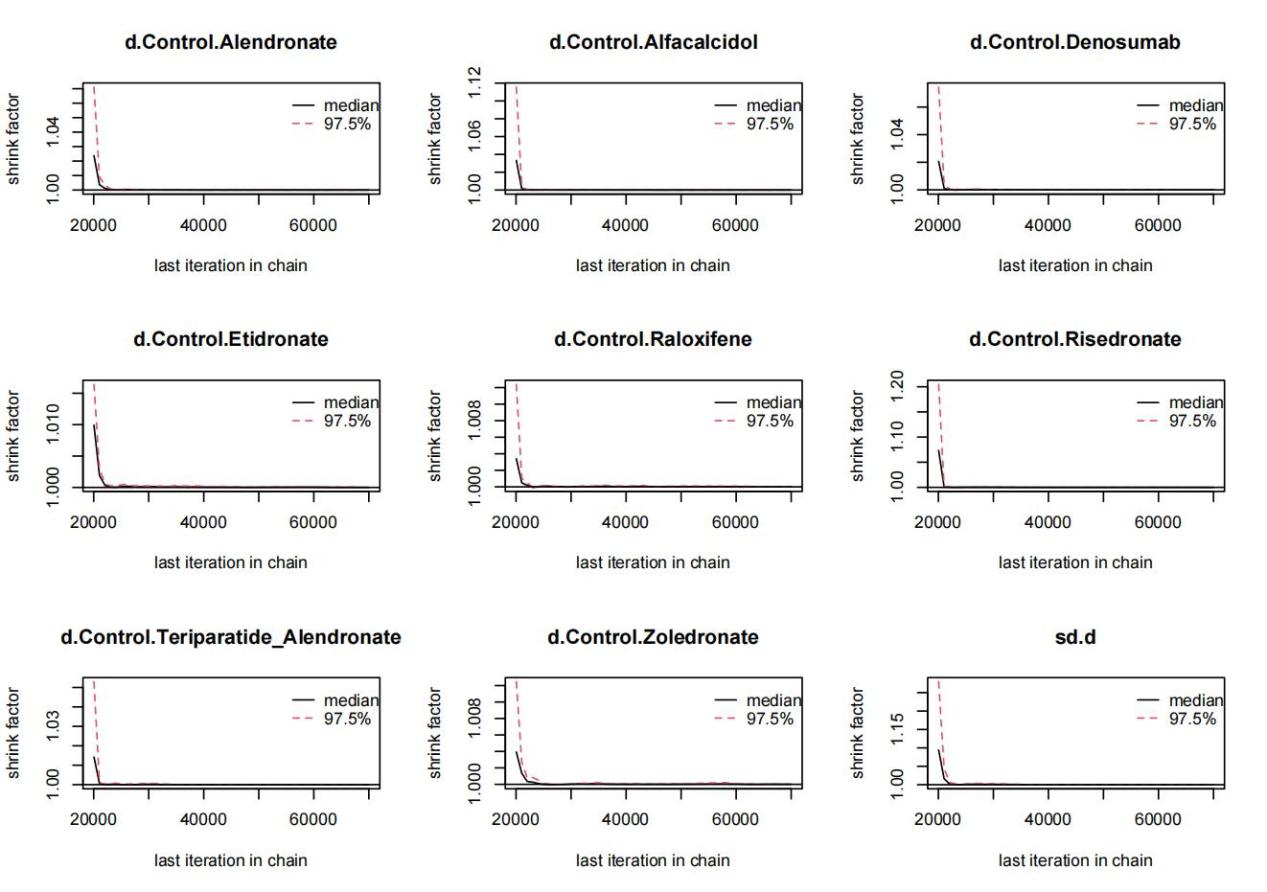


# Appendix 5 NMA result

## Figure 5.1: Individual study results grouped by treatment comparison (6 months)

**
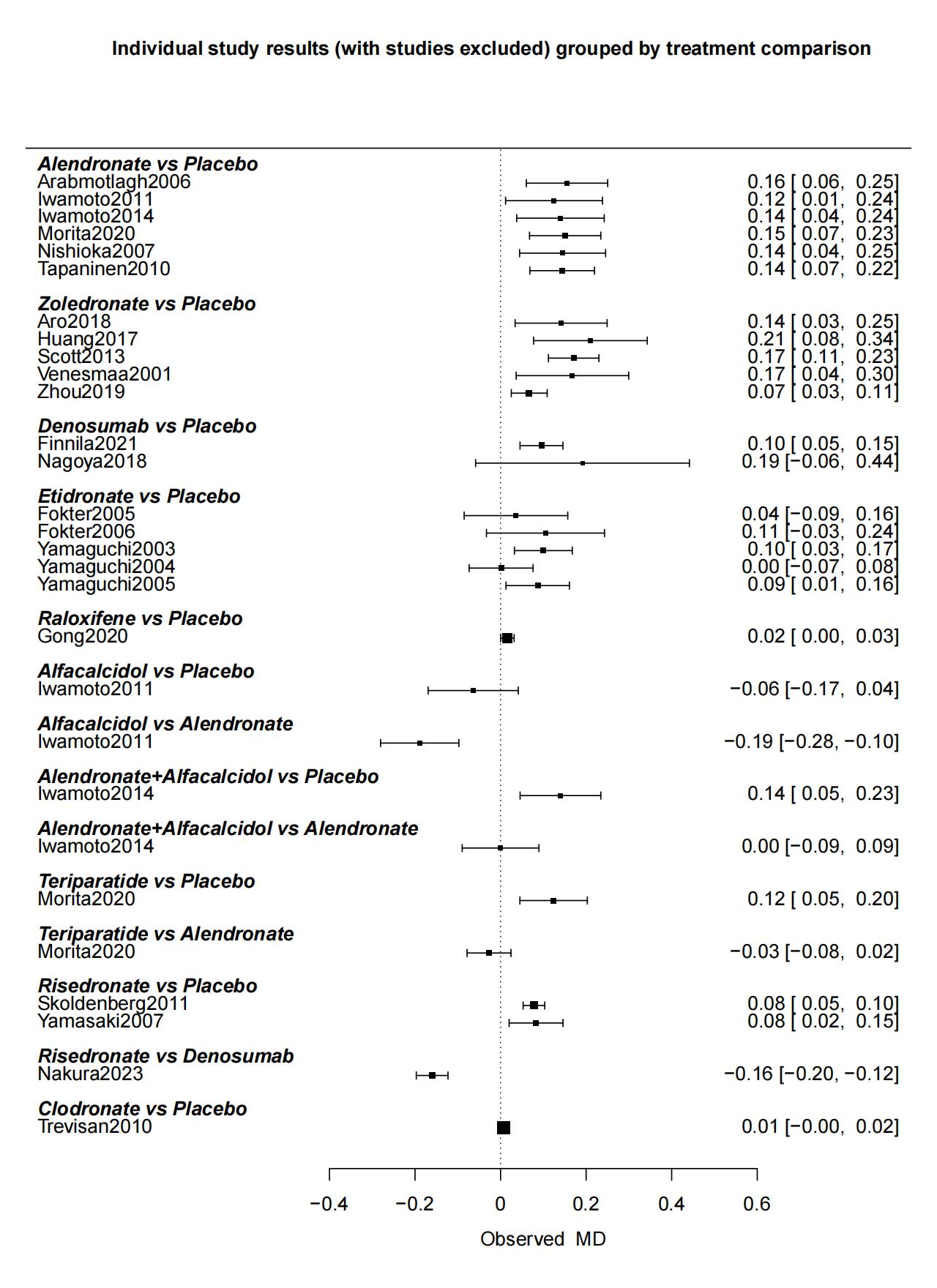
**

## Figure 5.2: Individual study results grouped by treatment comparison (12 months)

**
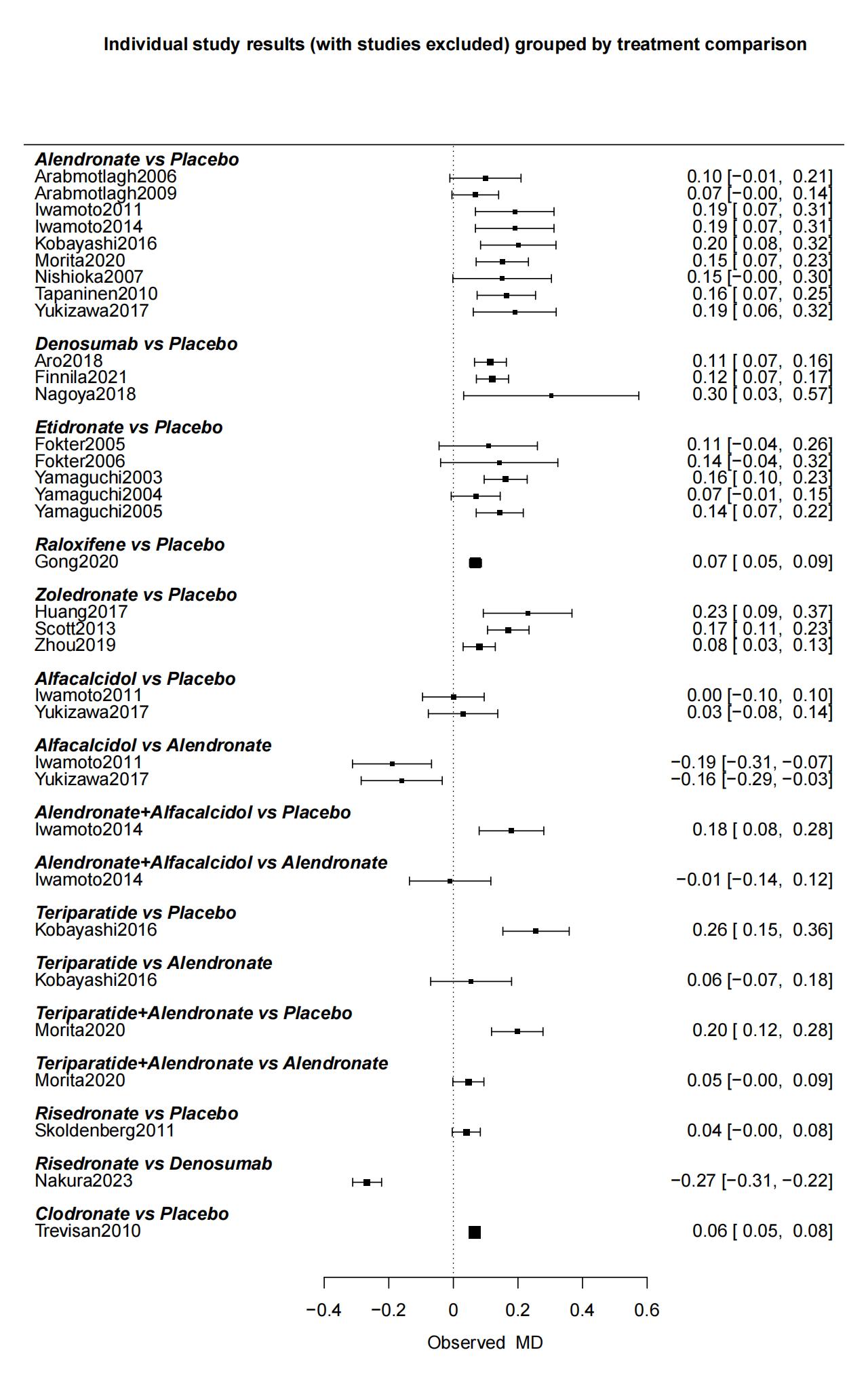
**

## Figure 5.3: Individual study results grouped by treatment comparison (24 months)

**
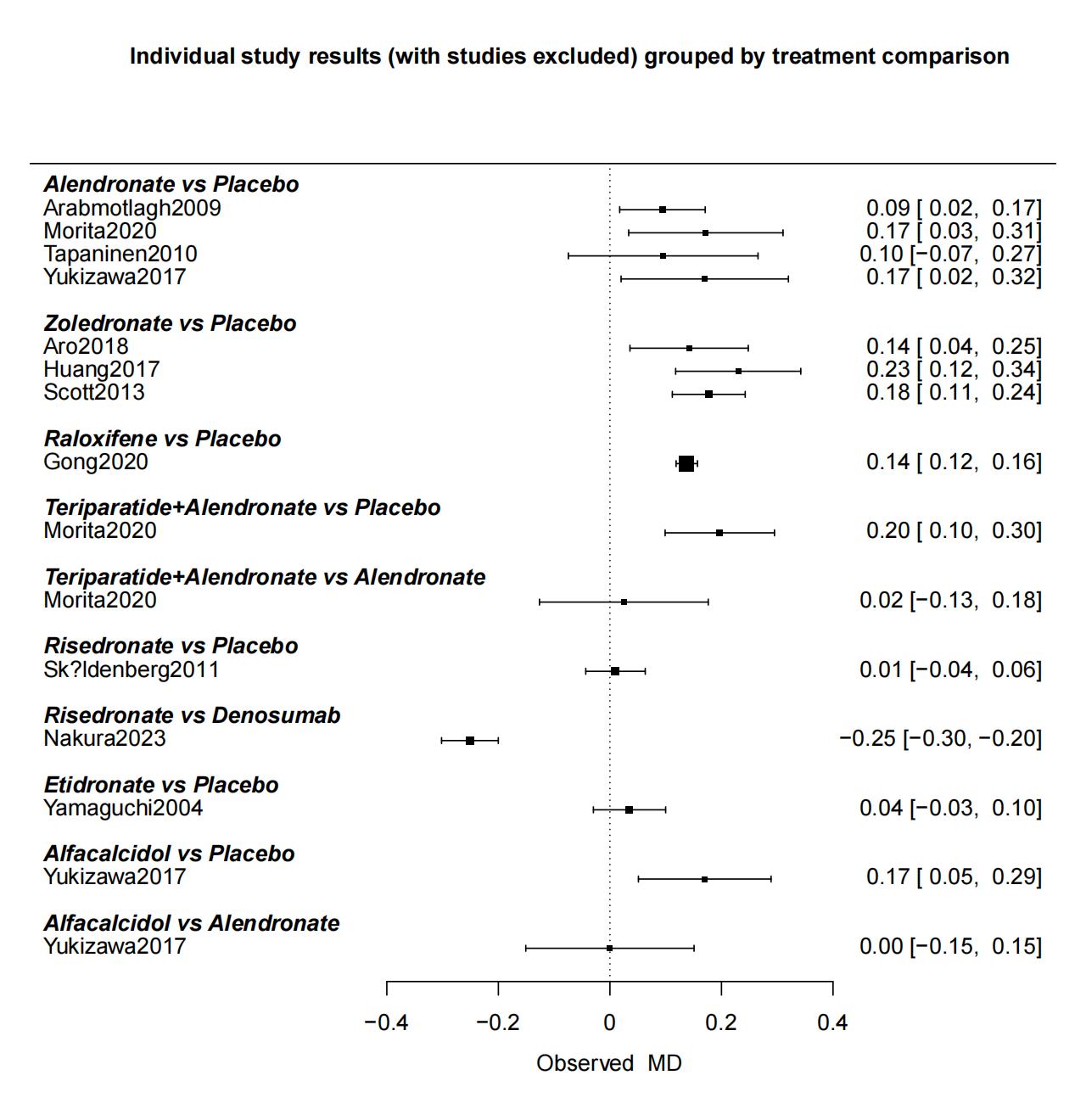
**

**Table 5.1: League Table (6 months)**

| **Alendronate** | - | - | - | - | - | - | - | - | - | - |
| --- | --- | --- | --- | --- | --- | --- | --- | --- | --- | --- |
| 0.11 (-0.92, 1.16) | **Alendronate + Alfacalcidol** | - | - | - | - | - | - | - | - | - |
| 1.29 (0.22, 2.38) | 1.18 (-0.27, 2.64) | **Alfacalcidol** | - | - | - | - | - | - | - | - |
| 0.8 (-0.37, 2.01) | 0.69 (-0.8, 2.19) | -0.49 (-2, 1.04) | **Clodronate** | - | - | - | - | - | - | - |
| -0.35 (-1.21, 0.59) | -0.46 (-1.7, 0.84) | -1.64 (-2.91, -0.31) | -1.15 (-2.43, 0.17) | **Denosumab** | - | - | - | - | - | - |
| 0.68 (-0.04, 1.43) | 0.57 (-0.58, 1.73) | -0.61 (-1.8, 0.59) | -0.12 (-1.31, 1.07) | 1.03 (0.11, 1.9) | **Etidronate** | - | - | - | - | - |
| 0.85 (-0.27, 2.02) | 0.74 (-0.7, 2.21) | -0.44 (-1.91, 1.06) | 0.05 (-1.43, 1.53) | 1.21 (-0.07, 2.43) | 0.17 (-0.97, 1.31) | **Raloxifene** | - | - | - | - |
| 0.51 (-0.34, 1.41) | 0.4 (-0.85, 1.67) | -0.78 (-2.05, 0.52) | -0.29 (-1.57, 0.99) | 0.86 (0.04, 1.65) | -0.17 (-1.03, 0.7) | -0.34 (-1.57, 0.9) | **Risedronate** | - | - | - |
| 0.1 (-0.95, 1.18) | -0.01 (-1.46, 1.43) | -1.19 (-2.66, 0.29) | -0.7 (-2.23, 0.8) | 0.45 (-0.88, 1.72) | -0.58 (-1.77, 0.6) | -0.75 (-2.23, 0.72) | -0.41 (-1.7, 0.86) | **Teriparatide** | - | - |
| 0.06 (-0.69, 0.82) | -0.05 (-1.22, 1.12) | -1.24 (-2.44, -0.02) | -0.75 (-1.95, 0.46) | 0.41 (-0.53, 1.29) | -0.63 (-1.38, 0.12) | -0.8 (-1.96, 0.35) | -0.45 (-1.35, 0.42) | -0.05 (-1.24, 1.16) | **Zoledronate** | - |
| 1.11 (0.61, 1.64) | 1 (-0.03, 2.04) | -0.18 (-1.25, 0.91) | 0.3 (-0.77, 1.39) | 1.46 (0.71, 2.17) | NA | NA | 0.6 (-0.11, 1.29) | 1.01 (-0.05, 2.08) | NA | **Control** |

**Table 5.2: League Table (12 months)**

| **Alendronate + Alfacalcidol** | - | - | - | - | - | - | - | - | - |
| --- | --- | --- | --- | --- | --- | --- | --- | --- | --- |
| 0.90 (-0.18, 1.99) | **Alfacalcidol** | - | - | - | - | - | - | - | - |
| -1.25 (-2.58, 0.09) | -2.16 (-3.36, -0.94) | **Clodronate** | - | - | - | - | - | - | - |
| -0.65 (-1.72, 0.48) | -1.55 (-2.46, -0.58) | 0.60 (-0.58, 1.83) | **Denosumab** | - | - | - | - | - | - |
| 0.20 (-0.78, 1.19) | -0.70 (-1.51, 0.10) | 1.45 (0.34, 2.55) | 0.85 (0.03, 1.61) | **Etidronate** | - | - | - | - | - |
| 0.01 (-1.19, 1.22) | -0.89 (-1.95, 0.18) | 1.26 (-0.03, 2.57) | 0.66 (-0.41, 1.69) | -0.19 (-1.1, 0.75) | **Raloxifene** | - | - | - | - |
| 1.15 (0.04, 2.33) | 0.25 (-0.72, 1.27) | 2.40 (1.18, 3.67) | 1.80 (1.05, 2.55) | 0.94 (0.13, 1.84) | 1.14 (0.07, 2.27) | **Risedronate** | - | - | - |
| -0.60 (-1.68, 0.48) | -1.50 (-2.43, -0.57) | 0.66 (-0.57, 1.86) | 0.06 (-0.93, 0.97) | -0.79 (-1.62, 0.01) | -0.60 (-1.69, 0.45) | -1.74 (-2.79, -0.78) | **Teriparatide** | - | - |
| 0.03 (-0.98, 1.03) | -0.88 (-1.71, -0.04) | 1.28 (0.15, 2.38) | 0.67 (-0.17, 1.47) | -0.17 (-0.84, 0.49) | 0.01 (-0.95, 0.97) | -1.12 (-2.04, -0.27) | 0.62 (-0.22, 1.47) | **Zoledronate** | - |
| 0.95 (0.08, 1.84) | 0.05 (-0.62, 0.73) | 2.20 (1.19, 3.21) | 1.60 (0.92, 2.24) | 0.75 (0.31,1.19) | 0.94 (0.12,1.76) | 0.19 (-0.50,0.96) | 1.55 (0.87, 2.24) | 0.93 (0.44,1.42) | **Control** |

**Table 5.3: League Table (24 months and over)**

| **Alendronate** | - | - | - | - | - | - | - | - |
| --- | --- | --- | --- | --- | --- | --- | --- | --- |
| -0.55 (-1.47, 0.35) | **Alfacalcidol** | - | - | - | - | - | - | - |
| -2.97 (-4.24, -1.71) | -2.42 (-3.89, -0.96) | **Denosumab** | - | - | - | - | - | - |
| -0.50 (-1.48, 0.47) | 0.05 (-1.18, 1.27) | 2.47 (1.04, 3.91) | **Etidronate** | - | - | - | - | - |
| -2.59 (-3.47, -1.70) | -2.03 (-3.18, -0.88) | 0.38 (-0.99, 1.77) | -2.09 (-3.21, -0.96) | **Raloxifene** | - | - | - | - |
| -0.86 (-1.79, 0.07) | -0.30 (-1.52, 0.88) | 2.11 (1.26, 2.96) | -0.36 (-1.52, 0.81) | 1.73 (0.64, 2.81) | **Risedronate** | - | - | - |
| -1.04 (-1.96, -0.15) | -0.49 (-1.72, 0.72) | 1.93 (0.45, 3.39) | -0.54 (-1.78, 0.69) | 1.55 (0.38, 2.70) | -0.19 (-1.38, 1.01) | **Teriparatide+Alendronate** | - | - |
| -1.85 (-2.56, -1.15) | -1.30 (-2.34, -0.27) | 1.12 (-0.16, 2.38) | -1.35 (-2.35, -0.37) | 0.73 (-0.17, 1.62) | -1.00 (-1.94, -0.05) | -0.81 (-1.84, 0.22) | **Zoledronate** | - |
| -0.77 (-1.27, -0.29) | -0.22 (-1.12, 0.68) | 2.19 (1.03, 3.36) | -0.28 (-1.12, 0.57) | 1.81 (1.07, 2.55) | 0.08 (-0.71, 0.89) | 0.26 (-0.63, 1.17) | 1.08 (0.58, 1.59) | **Control** |

# Appendix 6: Results of SIDE splitting results

## Table 6.1 Results of SIDE splitting results (6 months)

| **comparison** | **k** | **prop** | **nma** | **direct** | **indir.** | **Diff** | **z** | **p-value** |
| --- | --- | --- | --- | --- | --- | --- | --- | --- |
| Alendronate vs Alendronate_Alfacalcidol | 1 | 0.79 | 0.11 | 0 | 0.55 | -0.55 | -0.45 | 0.6509 |
| Alendronate vs Alfacalcidol | 1 | 0.79 | 1.3 | 1.09 | 2.1 | -1.02 | -0.79 | 0.4273 |
| Alendronate vs Clodronate | 0 | 0 | 0.81 | . | 0.81 | . | . | . |
| Alendronate vs Control | 6 | 1 | 1.11 | 1.11 | . | . | . | . |
| Alendronate vs Denosumab | 0 | 0 | -0.34 | . | -0.34 | . | . | . |
| Alendronate vs Etidronate | 0 | 0 | 0.68 | . | 0.68 | . | . | . |
| Alendronate vs Raloxifene | 0 | 0 | 0.85 | . | 0.85 | . | . | . |
| Alendronate vs Risedronate | 0 | 0 | 0.51 | . | 0.51 | . | . | . |
| Alendronate vs Teriparatide | 1 | 0.79 | 0.1 | 0.25 | -0.47 | 0.73 | 0.59 | 0.5579 |
| Alendronate vs Zoledronate | 0 | 0 | 0.06 | . | 0.06 | . | . | . |
| Alendronate + Alfacalcidol vs Alfacalcidol | 0 | 0 | 1.18 | . | 1.18 | . | . | . |
| Alendronate + Alfacalcidol vs Clodronate | 0 | 0 | 0.7 | . | 0.7 | . | . | . |
| Alendronate + Alfacalcidol vs Control | 1 | 0.8 | 1 | 0.89 | 1.44 | -0.55 | -0.45 | 0.6509 |
| Alendronate + Alfacalcidol vs Denosumab | 0 | 0 | -0.46 | . | -0.46 | . | . | . |
| Alendronate + Alfacalcidol vs Etidronate | 0 | 0 | 0.57 | . | 0.57 | . | . | . |
| Alendronate + Alfacalcidol vs Raloxifene | 0 | 0 | 0.74 | . | 0.74 | . | . | . |
| Alendronate + Alfacalcidol vs Risedronate | 0 | 0 | 0.4 | . | 0.4 | . | . | . |
| Alendronate + Alfacalcidol vs Teriparatide | 0 | 0 | -0.01 | . | -0.01 | . | . | . |
| Alendronate + Alfacalcidol vs Zoledronate | 0 | 0 | -0.06 | . | -0.06 | . | . | . |
| Alfacalcidol vs Clodronate | 0 | 0 | -0.49 | . | -0.49 | . | . | . |
| Alfacalcidol vs Control | 1 | 0.83 | -0.19 | -0.37 | 0.72 | -1.09 | -0.79 | 0.4273 |
| Alfacalcidol vs Denosumab | 0 | 0 | -1.64 | . | -1.64 | . | . | . |
| Alfacalcidol vs Etidronate | 0 | 0 | -0.61 | . | -0.61 | . | . | . |
| Alfacalcidol vs Raloxifene | 0 | 0 | -0.44 | . | -0.44 | . | . | . |
| Alfacalcidol vs Risedronate | 0 | 0 | -0.78 | . | -0.78 | . | . | . |
| Alfacalcidol vs Teriparatide | 0 | 0 | -1.19 | . | -1.19 | . | . | . |
| Alfacalcidol vs Zoledronate | 0 | 0 | -1.24 | . | -1.24 | . | . | . |
| Clodronate vs Control | 1 | 1 | 0.3 | 0.3 | . | . | . | . |
| Clodronate vs Denosumab | 0 | 0 | -1.15 | . | -1.15 | . | . | . |
| Clodronate vs Etidronate | 0 | 0 | -0.13 | . | -0.13 | . | . | . |
| Clodronate vs Raloxifene | 0 | 0 | 0.05 | . | 0.05 | . | . | . |
| Clodronate vs Risedronate | 0 | 0 | -0.29 | . | -0.29 | . | . | . |
| Clodronate vs Teriparatide | 0 | 0 | -0.71 | . | -0.71 | . | . | . |
| Clodronate vs Zoledronate | 0 | 0 | -0.75 | . | -0.75 | . | . | . |
| Denosumab vs Control | 2 | 0.71 | 1.45 | 0.85 | 2.96 | -2.11 | -2.78 | 0.0054 |
| Etidronate vs Control | 5 | 1 | 0.43 | 0.43 | . | . | . | . |
| Raloxifene vs Control | 1 | 1 | 0.26 | 0.26 | . | . | . | . |
| Risedronate vs Control | 2 | 0.75 | 0.6 | 1.12 | -0.99 | 2.11 | 2.78 | 0.0054 |
| Teriparatide vs Control | 1 | 0.77 | 1.01 | 1.17 | 0.46 | 0.71 | 0.59 | 0.5579 |
| Zoledronate vs Control | 5 | 1 | 1.05 | 1.05 | . | . | . | . |
| Denosumab vs Etidronate | 0 | 0 | 1.03 | . | 1.03 | . | . | . |
| Denosumab vs Raloxifene | 0 | 0 | 1.2 | . | 1.2 | . | . | . |
| Denosumab vs Risedronate | 1 | 0.53 | 0.86 | 1.84 | -0.27 | 2.11 | 2.78 | 0.0054 |
| Denosumab vs Teriparatide | 0 | 0 | 0.44 | . | 0.44 | . | . | . |
| Denosumab vs Zoledronate | 0 | 0 | 0.4 | . | 0.4 | . | . | . |
| Etidronate vs Raloxifene | 0 | 0 | 0.17 | . | 0.17 | . | . | . |
| Etidronate vs Risedronate | 0 | 0 | -0.17 | . | -0.17 | . | . | . |
| Etidronate vs Teriparatide | 0 | 0 | -0.58 | . | -0.58 | . | . | . |
| Etidronate vs Zoledronate | 0 | 0 | -0.63 | . | -0.63 | . | . | . |
| Raloxifene vs Risedronate | 0 | 0 | -0.34 | . | -0.34 | . | . | . |
| Raloxifene vs Teriparatide | 0 | 0 | -0.75 | . | -0.75 | . | . | . |
| Raloxifene vs Zoledronate | 0 | 0 | -0.8 | . | -0.8 | . | . | . |
| Risedronate vs Teriparatide | 0 | 0 | -0.41 | . | -0.41 | . | . | . |
| Risedronate vs Zoledronate | 0 | 0 | -0.46 | . | -0.46 | . | . | . |
| Teriparatide vs Zoledronate | 0 | 0 | -0.05 | . | -0.05 | . | . | . |

## Table 6.2 Details of SIDE splitting results (12 months)

| **comparison** | **k** | **prop** | **nma** | **direct** | **indir.** | **Diff** | **z** | **p-value** |
| --- | --- | --- | --- | --- | --- | --- | --- | --- |
| Alendronate vs Alendronate + Alfacalcidol | 1 | 0.78 | 0.05 | 0.05 | 0.03 | 0.02 | 0.02 | 0.9863 |
| Alendronate vs Alfacalcidol | 2 | 0.81 | 0.95 | 0.97 | 0.88 | 0.09 | 0.09 | 0.931 |
| Alendronate vs Control | 9 | 1 | 1.01 | 1.01 | . | . | . | . |
| Alendronate vs Denosumab | 0 | 0 | -0.85 | . | -0.85 | . | . | . |
| Alendronate vs Etidronate | 0 | 0 | 0.26 | . | 0.26 | . | . | . |
| Alendronate vs Raloxifene | 0 | 0 | 0.07 | . | 0.07 | . | . | . |
| Alendronate vs Risedronate | 0 | 0 | 0.4 | . | 0.4 | . | . | . |
| Alendronate vs Teriparatide | 2 | 0.82 | -0.56 | -0.4 | -1.3 | 0.9 | 0.82 | 0.4145 |
| Alendronate vs Zoledronate | 0 | 0 | 0.08 | . | 0.08 | . | . | . |
| Alendronate + Alfacalcidol vs Alfacalcidol | 0 | 0 | 0.9 | . | 0.9 | . | . | . |
| Alendronate + Alfacalcidol vs Control | 1 | 0.78 | 0.97 | 0.97 | 0.95 | 0.02 | 0.02 | 0.9863 |
| Alendronate + Alfacalcidol vs Denosumab | 0 | 0 | -0.9 | . | -0.9 | . | . | . |
| Alendronate + Alfacalcidol vs Etidronate | 0 | 0 | 0.21 | . | 0.21 | . | . | . |
| Alendronate + Alfacalcidol vs Raloxifene | 0 | 0 | 0.02 | . | 0.02 | . | . | . |
| Alendronate + Alfacalcidol vs Risedronate | 0 | 0 | 0.35 | . | 0.35 | . | . | . |
| Alendronate + Alfacalcidol vs Teriparatide | 0 | 0 | -0.61 | . | -0.61 | . | . | . |
| Alendronate + Alfacalcidol vs Zoledronate | 0 | 0 | 0.03 | . | 0.03 | . | . | . |
| Alfacalcidol vs Control | 2 | 0.82 | 0.06 | 0.08 | -0.02 | 0.1 | 0.09 | 0.9317 |
| Alfacalcidol vs Denosumab | 0 | 0 | -1.8 | . | -1.8 | . | . | . |
| Alfacalcidol vs Etidronate | 0 | 0 | -0.7 | . | -0.7 | . | . | . |
| Alfacalcidol vs Raloxifene | 0 | 0 | -0.88 | . | -0.88 | . | . | . |
| Alfacalcidol vs Risedronate | 0 | 0 | -0.55 | . | -0.55 | . | . | . |
| Alfacalcidol vs Teriparatide | 0 | 0 | -1.51 | . | -1.51 | . | . | . |
| Alfacalcidol vs Zoledronate | 0 | 0 | -0.87 | . | -0.87 | . | . | . |
| Denosumab vs Control | 2 | 0.72 | 1.86 | 1.13 | 3.79 | -2.66 | -2.96 | 0.0031 |
| Etidronate vs Control | 5 | 1 | 0.76 | 0.76 | . | . | . | . |
| Raloxifene vs Control | 1 | 1 | 0.94 | 0.94 | . | . | . | . |
| Risedronate vs Control | 2 | 0.76 | 0.62 | 1.25 | -1.42 | 2.66 | 2.96 | 0.0031 |
| Teriparatide vs Control | 2 | 0.78 | 1.57 | 1.76 | 0.91 | 0.85 | 0.82 | 0.4096 |
| Zoledronate vs Control | 4 | 1 | 0.94 | 0.94 | . | . | . | . |
| Denosumab vs Etidronate | 0 | 0 | 1.11 | . | 1.11 | . | . | . |
| Denosumab vs Raloxifene | 0 | 0 | 0.92 | . | 0.92 | . | . | . |
| Denosumab vs Risedronate | 1 | 0.51 | 1.25 | 2.54 | -0.12 | 2.66 | 2.96 | 0.0031 |
| Denosumab vs Teriparatide | 0 | 0 | 0.29 | . | 0.29 | . | . | . |
| Denosumab vs Zoledronate | 0 | 0 | 0.93 | . | 0.93 | . | . | . |
| Etidronate vs Raloxifene | 0 | 0 | -0.19 | . | -0.19 | . | . | . |
| Etidronate vs Risedronate | 0 | 0 | 0.14 | . | 0.14 | . | . | . |
| Etidronate vs Teriparatide | 0 | 0 | -0.82 | . | -0.82 | . | . | . |
| Etidronate vs Zoledronate | 0 | 0 | -0.18 | . | -0.18 | . | . | . |
| Raloxifene vs Risedronate | 0 | 0 | 0.33 | . | 0.33 | . | . | . |
| Raloxifene vs Teriparatide | 0 | 0 | -0.63 | . | -0.63 | . | . | . |
| Raloxifene vs Zoledronate | 0 | 0 | 0.01 | . | 0.01 | . | . | . |
| Risedronate vs Teriparatide | 0 | 0 | -0.96 | . | -0.96 | . | . | . |
| Risedronate vs Zoledronate | 0 | 0 | -0.32 | . | -0.32 | . | . | . |
| Teriparatide vs Zoledronate | 0 | 0 | 0.64 | . | 0.64 | . | . | . |

## Table 6.3 Details of SIDE splitting results (24 months and over)

| **comparison** | **k** | **prop** | **nma** | **direct** | **indir.** | **Diff** | **z** | **p-value** |
| --- | --- | --- | --- | --- | --- | --- | --- | --- |
| Alendronate vs Alfacalcidol | 1 | 0.8 | 0.75 | 0.23 | 2.76 | -2.53 | -2.01 | 0.04 |
| Alendronate vs Control | 4 | 1 | 0.43 | 0.43 | . | . | . | . |
| Alendronate vs Denosumab | 0 | 0 | -1.77 | . | -1.77 | . | . | . |
| Alendronate vs Etidronate | 0 | 0 | 0.15 | . | 0.15 | . | . | . |
| Alendronate vs Raloxifene | 0 | 0 | -1.39 | . | -1.39 | . | . | . |
| Alendronate vs Risedronate | 0 | 0 | 0.34 | . | 0.34 | . | . | . |
| Alendronate vs Teriparatide + Alendronate | 1 | 0.81 | -0.44 | -0.15 | -1.69 | 1.54 | 1.16 | 0.25 |
| Alendronate vs Zoledronate | 0 | 0 | -0.66 | . | -0.66 | . | . | . |
| Alfacalcidol vs Control | 1 | 0.82 | -0.32 | -0.79 | 1.84 | -2.63 | -2.01 | 0.04 |
| Alfacalcidol vs Denosumab | 0 | 0 | -2.52 | . | -2.52 | . | . | . |
| Alfacalcidol vs Etidronate | 0 | 0 | -0.60 | . | -0.60 | . | . | . |
| Alfacalcidol vs Raloxifene | 0 | 0 | -2.13 | . | -2.13 | . | . | . |
| Alfacalcidol vs Risedronate | 0 | 0 | -0.40 | . | -0.40 | . | . | . |
| Alfacalcidol vs Teriparatide + Alendronate | 0 | 0 | -1.19 | . | -1.19 | . | . | . |
| Alfacalcidol vs Zoledronate | 0 | 0 | -1.40 | . | -1.40 | . | . | . |
| Denosumab vs Control | 0 | 0 | 2.20 | . | 2.20 | . | . | . |
| Etidronate vs Control | 1 | 1 | 0.28 | 0.28 | . | . | . | . |
| Raloxifene vs Control | 1 | 1 | 1.81 | 1.81 | . | . | . | . |
| Risedronate vs Control | 1 | 1 | 0.08 | 0.08 | . | . | . | . |
| Teriparatide + Alendronate vs Control | 1 | 0.8 | 0.87 | 1.17 | -0.36 | 1.53 | 1.16 | 0.25 |
| Zoledronate vs Control | 3 | 1 | 1.09 | 1.09 | . | . | . | . |
| Denosumab vs Etidronate | 0 | 0 | 1.92 | . | 1.92 | . | . | . |
| Denosumab vs Raloxifene | 0 | 0 | 0.39 | . | 0.39 | . | . | . |
| Denosumab vs Risedronate | 1 | 1 | 2.12 | 2.12 | . | . | . | . |
| vs Teriparatide + Alendronate | 0 | 0 | 1.33 | . | 1.33 | . | . | . |
| Denosumab vs Zoledronate | 0 | 0 | 1.11 | . | 1.11 | . | . | . |
| Etidronate vs Raloxifene | 0 | 0 | -1.53 | . | -1.53 | . | . | . |
| Etidronate vs Risedronate | 0 | 0 | 0.19 | . | 0.19 | . | . | . |
| Etidronate vs Teriparatide + Alendronate | 0 | 0 | -0.59 | . | -0.59 | . | . | . |
| Etidronate vs Zoledronate | 0 | 0 | -0.81 | . | -0.81 | . | . | . |
| Raloxifene vs Risedronate | 0 | 0 | 1.73 | . | 1.73 | . | . | . |
| Raloxifene vs Teriparatide + Alendronate | 0 | 0 | 0.94 | . | 0.94 | . | . | . |
| Raloxifene vs Zoledronate | 0 | 0 | 0.73 | . | 0.73 | . | . | . |
| Risedronate vs Teriparatide + Alendronate | 0 | 0 | -0.79 | . | -0.79 | . | . | . |
| Risedronate vs Zoledronate | 0 | 0 | -1.00 | . | -1.00 | . | . | . |
| Teriparatide + Alendronate vs Zoledronate | 0 | 0 | -0.22 | . | -0.22 | . | . | . |

*Legend:*

*comparison - Treatment comparison*

*k - Number of studies providing direct evidence*

*prop - Direct evidence proportion*

*nma - Estimated treatment effect (SMD) in network meta-analysis*

*direct - Estimated treatment effect (SMD) derived from direct evidence*

*indir. - Estimated treatment effect (SMD) derived from indirect evidence*

*Diff - Difference between direct and indirect treatment estimates*

*z - z-value of test for disagreement (direct versus indirect)*

*p-value - p-value of test for disagreement (direct versus indirect)*

# Appendix 7: Sensitivity analyses

## Table 7.1 Changes in heterogeneity

We assessed the sensitivity of our findings by repeating each NMA after excluding studies at overall high risk of bias. Below we present the results from the changes in heterogeneity in each sensitivity analysis.

| **Months** | **Including only studies with** | **Number of studies included** | **I^2^** | **Change** |
| --- | --- | --- | --- | --- |
| **6months** | None | 23 | 57.11 | - |
|  | studies at overall low to moderate risk of bias | 17 | 69.06 | 20.92% |
| **12months** | None | 25 | 38.36 | - |
|  | studies at overall low to moderate risk of bias | 19 | 37.36 | -2.61% |
| **24months and over** | None | 11 | <0.001 | - |
|  | studies at overall low to moderate risk of bias | 10 | 3.64 | - |

## Figure 7.1 Exclude studies at overall high risk of bias (6 months)

After excluding trials with high overall risk of bias, the hierarchy did not change significantly.


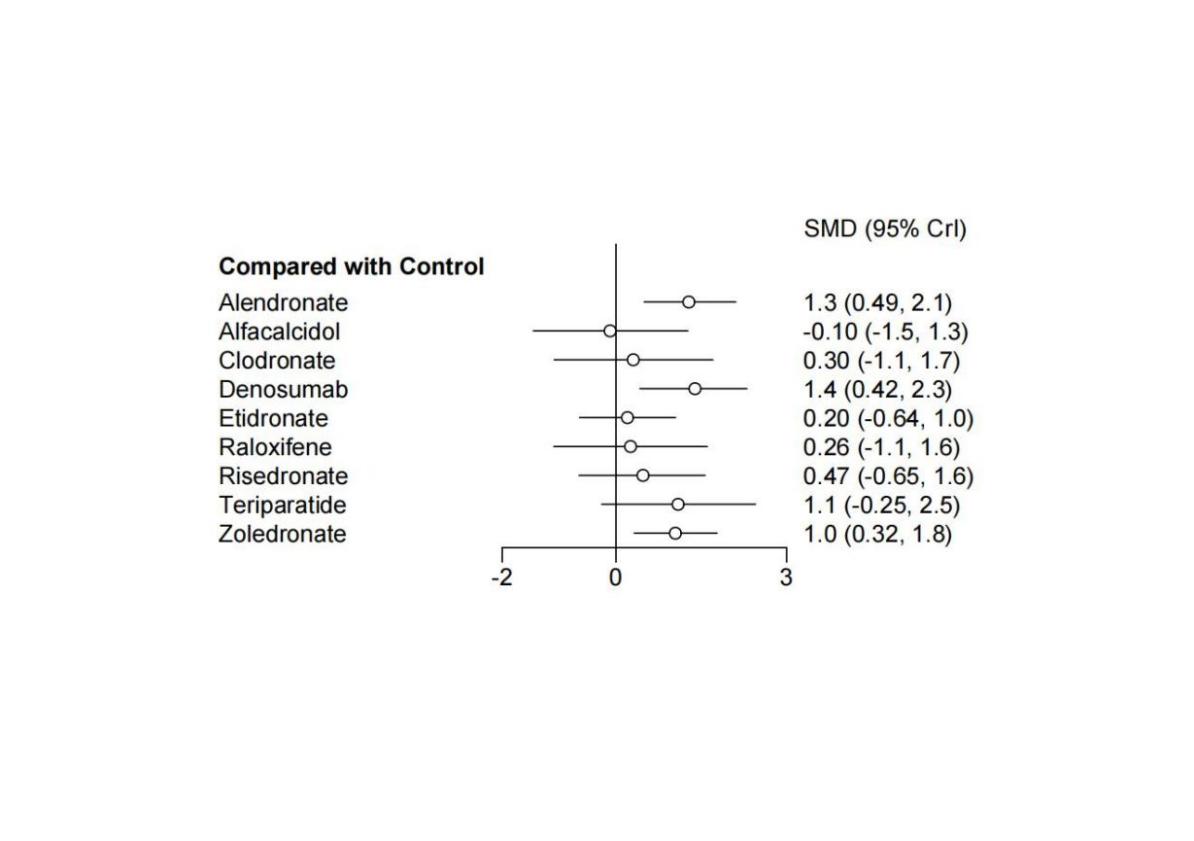


## Figure 7.2 Exclude studies at overall high risk of bias (12 months)

After excluding trials with high overall risk of bias, the hierarchy did not change significantly.


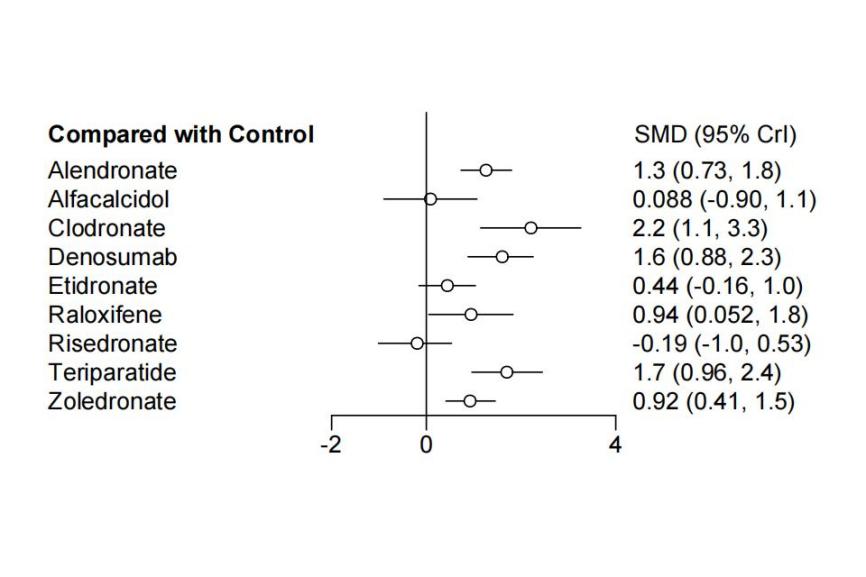


## Figure 7.3 Exclude studies at overall high risk of bias (24 months and over)

After excluding trials with high overall risk of bias, the hierarchy did not change significantly.


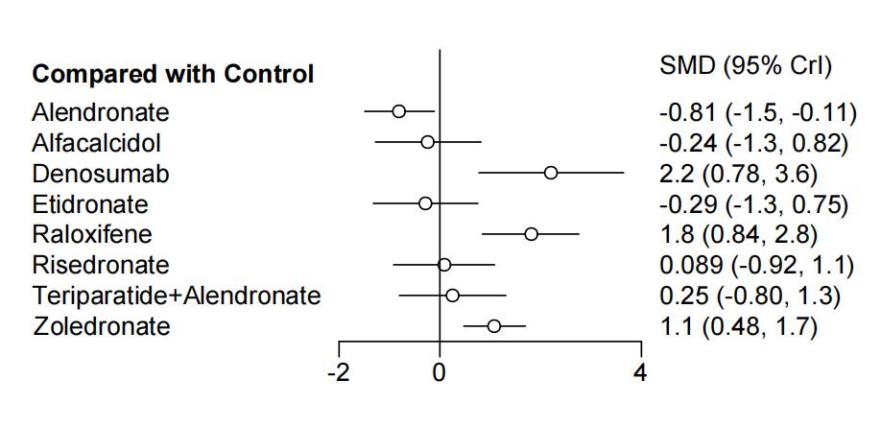


# Appendix 8 Confidence in Network Meta-Analysis (CINeMA)

In CINeMA's comparative network plots of randomized controlled trials, dots represent different interventions and lines represent direct comparisons. The colors of the dots and lines indicate risk of deviation: low (green), medium (yellow), and high (red). The CINeMA table details the risk of bias for different comparisons.

**Figure 8.1 CINeMA netplot (6 months)**

**
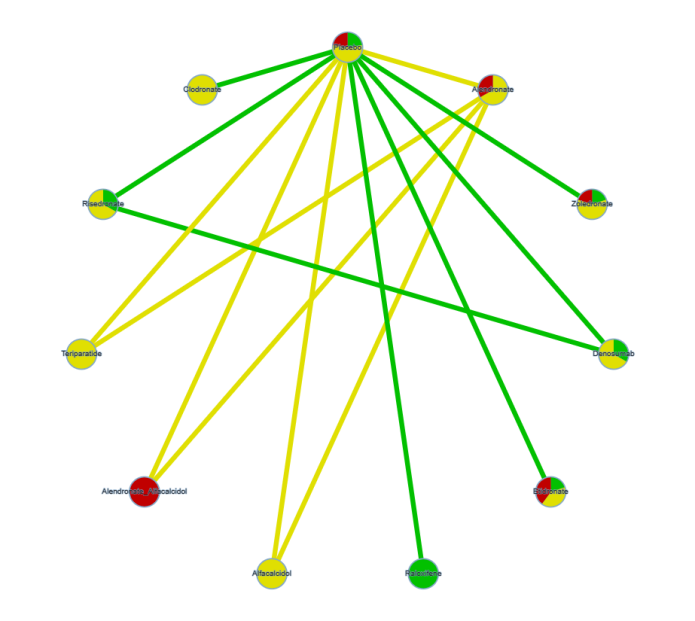
**

**Figure 8.2 CINeMA netplot (12 months)**

**
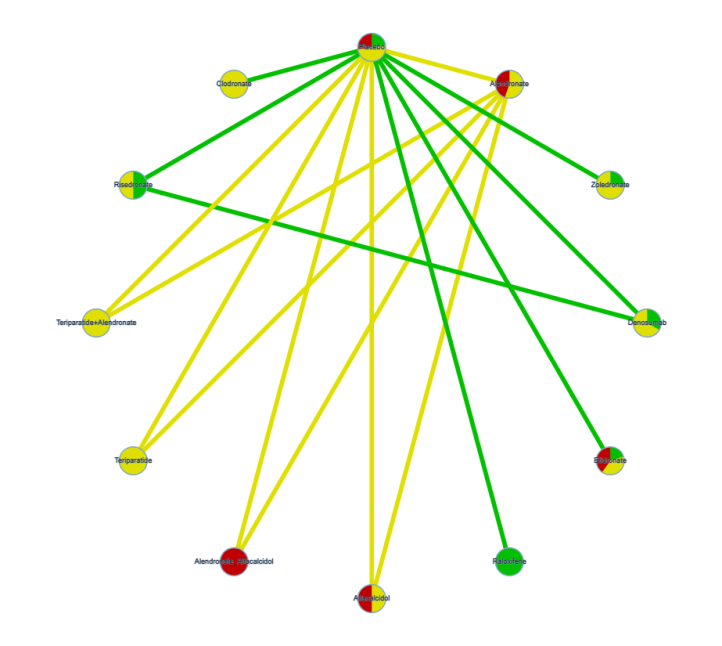
**

**Figure 8.3 CINeMA netplot (24 months and over)**

**
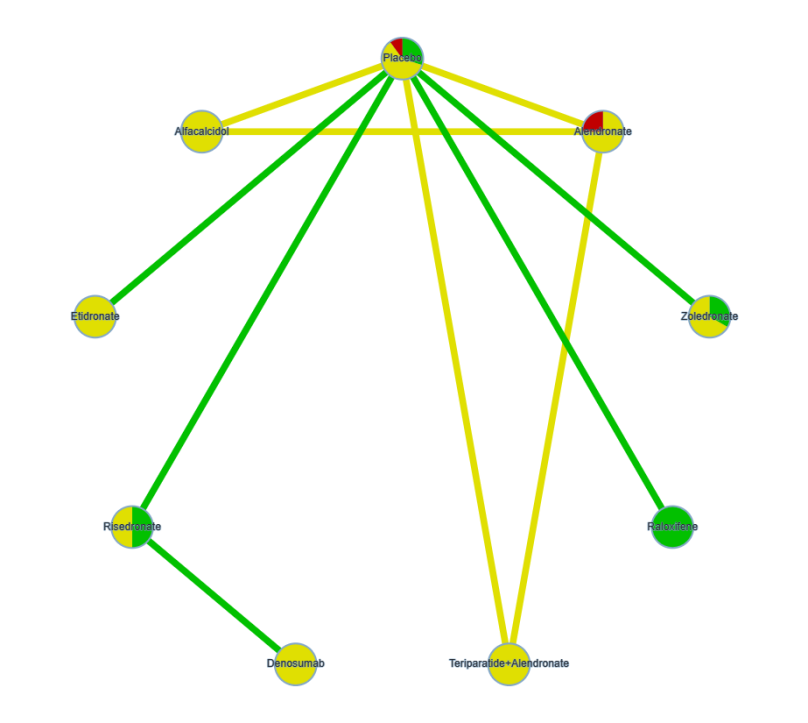
**

**Table 8.1 CINeMA report (6 months)**

| **Comparison** | **Number of studies** | **Within-study bias** | **Reporting bias** | **Indirectness** | **Imprecision** | **Heterogeneity** | **Incoherence** | **Confidence rating** | **Reason(s) for downgrading** |
| --- | --- | --- | --- | --- | --- | --- | --- | --- | --- |
| Alendronate:Alendronate_Alfacalcidol | 1 | Major concerns | Low risk | Some concerns | No concerns | No concerns | No concerns | Low | ["Within-study bias"] |
| Alendronate:Alfacalcidol | 1 | Some concerns | Low risk | Some concerns | No concerns | Some concerns | No concerns | Moderate | - |
| Alendronate:Placebo | 6 | Some concerns | Low risk | Some concerns | No concerns | Some concerns | Major concerns | Low | ["Incoherence"] |
| Alendronate:Teriparatide | 1 | Some concerns | Low risk | Some concerns | No concerns | No concerns | No concerns | Moderate | - |
| Alendronate_Alfacalcidol:Placebo | 1 | Major concerns | Low risk | Some concerns | No concerns | Some concerns | No concerns | Low | ["Within-study bias"] |
| Alfacalcidol:Placebo | 1 | Some concerns | Low risk | Some concerns | No concerns | No concerns | No concerns | Moderate | - |
| Clodronate:Placebo | 1 | Some concerns | Low risk | No concerns | No concerns | No concerns | Major concerns | Low | ["Incoherence"] |
| Denosumab:Placebo | 2 | No concerns | Low risk | No concerns | No concerns | No concerns | Some concerns | High | - |
| Denosumab:Risedronate | 1 | Some concerns | Low risk | No concerns | No concerns | Some concerns | Some concerns | High | - |
| Etidronate:Placebo | 5 | Major concerns | Low risk | No concerns | No concerns | No concerns | Major concerns | Very low | ["Within-study bias"] |
| Placebo:Raloxifene | 1 | No concerns | Low risk | No concerns | No concerns | No concerns | Major concerns | Low | - |
| Placebo:Risedronate | 2 | Some concerns | Low risk | No concerns | No concerns | No concerns | Some concerns | High | - |
| Placebo:Teriparatide | 1 | Some concerns | Low risk | Some concerns | No concerns | Some concerns | No concerns | Moderate | - |
| Placebo:Zoledronate | 5 | Some concerns | Low risk | No concerns | No concerns | Some concerns | Major concerns | Low | ["Incoherence"] |
| Alendronate:Clodronate | 0 | Some concerns | Low risk | No concerns | No concerns | Some concerns | No concerns | Moderate | - |
| Alendronate:Denosumab | 0 | Some concerns | Low risk | No concerns | No concerns | No concerns | No concerns | Moderate | - |
| Alendronate:Etidronate | 0 | Some concerns | Low risk | No concerns | No concerns | No concerns | No concerns | High | - |
| Alendronate:Raloxifene | 0 | No concerns | Low risk | No concerns | No concerns | Some concerns | No concerns | Moderate | - |
| Alendronate:Risedronate | 0 | Some concerns | Low risk | No concerns | No concerns | No concerns | No concerns | High | - |
| Alendronate:Zoledronate | 0 | Some concerns | Low risk | No concerns | No concerns | No concerns | No concerns | High | - |
| Alendronate_Alfacalcidol:Alfacalcidol | 0 | Some concerns | Low risk | Some concerns | Some concerns | No concerns | No concerns | Moderate | - |
| Alendronate_Alfacalcidol:Clodronate | 0 | Some concerns | Low risk | Some concerns | Some concerns | No concerns | No concerns | Moderate | - |
| Alendronate_Alfacalcidol:Denosumab | 0 | Major concerns | Low risk | No concerns | No concerns | Some concerns | No concerns | Low | ["Within-study bias"] |
| Alendronate_Alfacalcidol:Etidronate | 0 | Major concerns | Low risk | Some concerns | No concerns | Some concerns | No concerns | Low | ["Within-study bias"] |
| Alendronate_Alfacalcidol:Raloxifene | 0 | Major concerns | Low risk | Some concerns | Some concerns | No concerns | No concerns | Low | ["Within-study bias"] |
| Alendronate_Alfacalcidol:Risedronate | 0 | Major concerns | Low risk | No concerns | No concerns | No concerns | No concerns | Low | ["Within-study bias"] |
| Alendronate_Alfacalcidol:Teriparatide | 0 | Some concerns | Low risk | Some concerns | No concerns | No concerns | No concerns | High | - |
| Alendronate_Alfacalcidol:Zoledronate | 0 | Major concerns | Low risk | Some concerns | No concerns | No concerns | No concerns | Low | ["Within-study bias"] |
| Alfacalcidol:Clodronate | 0 | Some concerns | Low risk | Some concerns | No concerns | Some concerns | No concerns | Moderate | - |
| Alfacalcidol:Denosumab | 0 | Some concerns | Low risk | No concerns | No concerns | No concerns | No concerns | High | - |
| Alfacalcidol:Etidronate | 0 | Some concerns | Low risk | Some concerns | No concerns | Some concerns | No concerns | Moderate | - |
| Alfacalcidol:Raloxifene | 0 | Some concerns | Low risk | Some concerns | No concerns | Some concerns | No concerns | Moderate | - |
| Alfacalcidol:Risedronate | 0 | Some concerns | Low risk | No concerns | No concerns | Some concerns | No concerns | High | - |
| Alfacalcidol:Teriparatide | 0 | Some concerns | Low risk | Some concerns | Some concerns | No concerns | No concerns | Moderate | - |
| Alfacalcidol:Zoledronate | 0 | Some concerns | Low risk | Some concerns | No concerns | Some concerns | No concerns | Moderate | - |
| Clodronate:Denosumab | 0 | Some concerns | Low risk | No concerns | Some concerns | No concerns | No concerns | High | - |
| Clodronate:Etidronate | 0 | Some concerns | Low risk | No concerns | No concerns | No concerns | No concerns | High | - |
| Clodronate:Raloxifene | 0 | No concerns | Low risk | No concerns | No concerns | No concerns | No concerns | High | - |
| Clodronate:Risedronate | 0 | Some concerns | Low risk | No concerns | No concerns | No concerns | No concerns | High | - |
| Clodronate:Teriparatide | 0 | Some concerns | Low risk | Some concerns | Some concerns | No concerns | No concerns | Moderate | - |
| Clodronate:Zoledronate | 0 | Some concerns | Low risk | No concerns | No concerns | Some concerns | No concerns | High | - |
| Denosumab:Etidronate | 0 | Some concerns | Low risk | No concerns | No concerns | Some concerns | No concerns | High | - |
| Denosumab:Raloxifene | 0 | No concerns | Low risk | No concerns | No concerns | Some concerns | No concerns | High | - |
| Denosumab:Teriparatide | 0 | Some concerns | Low risk | No concerns | No concerns | Some concerns | No concerns | High | - |
| Denosumab:Zoledronate | 0 | Some concerns | Low risk | No concerns | No concerns | No concerns | No concerns | High | - |
| Etidronate:Raloxifene | 0 | No concerns | Low risk | No concerns | No concerns | No concerns | No concerns | High | - |
| Etidronate:Risedronate | 0 | Some concerns | Low risk | No concerns | No concerns | No concerns | No concerns | High | - |
| Etidronate:Teriparatide | 0 | Some concerns | Low risk | Some concerns | No concerns | Some concerns | No concerns | Moderate | - |
| Etidronate:Zoledronate | 0 | Some concerns | Low risk | No concerns | No concerns | No concerns | No concerns | High | - |
| Raloxifene:Risedronate | 0 | No concerns | Low risk | No concerns | No concerns | No concerns | No concerns | High | - |
| Raloxifene:Teriparatide | 0 | Some concerns | Low risk | Some concerns | Some concerns | No concerns | No concerns | Moderate | - |
| Raloxifene:Zoledronate | 0 | No concerns | Low risk | No concerns | No concerns | Some concerns | No concerns | High | - |
| Risedronate:Teriparatide | 0 | Some concerns | Low risk | No concerns | No concerns | No concerns | No concerns | High | - |
| Risedronate:Zoledronate | 0 | Some concerns | Low risk | No concerns | No concerns | No concerns | No concerns | High | - |
| Teriparatide:Zoledronate | 0 | Some concerns | Low risk | Some concerns | No concerns | No concerns | No concerns | Moderate | - |

**Table 8.2 CINeMA report (12 months)**

| Comparison | Number of studies | Within-study bias | Reporting bias | Indirectness | Imprecision | Heterogeneity | Incoherence | Confidence rating | Reason(s) for downgrading |
| --- | --- | --- | --- | --- | --- | --- | --- | --- | --- |
| Alendronate:Alendronate_Alfacalcidol | 1 | Major concerns | Low risk | Some concerns | No concerns | No concerns | No concerns | Low | ["Within-study bias"] |
| Alendronate:Alfacalcidol | 2 | Major concerns | Low risk | Some concerns | No concerns | Some concerns | No concerns | Low | ["Within-study bias"] |
| Alendronate:Placebo | 8 | Major concerns | Low risk | Some concerns | No concerns | No concerns | No concerns | Low | ["Within-study bias"] |
| Alendronate:Teriparatide | 1 | Some concerns | Low risk | Some concerns | No concerns | No concerns | No concerns | Moderate | - |
| Alendronate:Teriparatide+Alendronate | 1 | Some concerns | Low risk | Some concerns | No concerns | Some concerns | No concerns | Moderate | - |
| Alendronate_Alfacalcidol:Placebo | 1 | Major concerns | Low risk | Some concerns | No concerns | Some concerns | No concerns | Low | ["Within-study bias"] |
| Alfacalcidol:Placebo | 1 | Major concerns | Low risk | Some concerns | No concerns | No concerns | No concerns | Low | ["Within-study bias"] |
| Clodronate:Placebo | 1 | Some concerns | Low risk | No concerns | No concerns | No concerns | Some concerns | Moderate | - |
| Denosumab:Placebo | 2 | No concerns | Low risk | No concerns | No concerns | No concerns | Some concerns | High | - |
| Denosumab:Risedronate | 1 | Some concerns | Low risk | No concerns | No concerns | No concerns | Some concerns | Moderate | - |
| Etidronate:Placebo | 5 | Some concerns | Low risk | No concerns | No concerns | No concerns | Some concerns | Moderate | - |
| Placebo:Raloxifene | 1 | No concerns | Low risk | No concerns | No concerns | Some concerns | Some concerns | Moderate | - |
| Placebo:Risedronate | 1 | No concerns | Low risk | No concerns | No concerns | No concerns | Some concerns | High | - |
| Placebo:Teriparatide | 1 | Some concerns | Low risk | Some concerns | No concerns | No concerns | No concerns | High | - |
| Placebo:Teriparatide+Alendronate | 1 | Some concerns | Low risk | Some concerns | No concerns | No concerns | No concerns | Moderate | - |
| Placebo:Zoledronate | 4 | Some concerns | Low risk | No concerns | No concerns | No concerns | Some concerns | Moderate | - |
| Alendronate:Clodronate | 0 | Some concerns | Low risk | No concerns | No concerns | Some concerns | Some concerns | Moderate | - |
| Alendronate:Denosumab | 0 | Some concerns | Low risk | No concerns | No concerns | No concerns | Some concerns | Moderate | - |
| Alendronate:Etidronate | 0 | Some concerns | Low risk | No concerns | No concerns | No concerns | Some concerns | High | - |
| Alendronate:Raloxifene | 0 | Some concerns | Low risk | No concerns | No concerns | No concerns | Some concerns | High | - |
| Alendronate:Risedronate | 0 | Some concerns | Low risk | No concerns | No concerns | Some concerns | Some concerns | Moderate | - |
| Alendronate:Zoledronate | 0 | Some concerns | Low risk | No concerns | No concerns | No concerns | Some concerns | High | - |
| Alendronate_Alfacalcidol:Alfacalcidol | 0 | Major concerns | Low risk | Some concerns | Some concerns | No concerns | Some concerns | Low | ["Within-study bias"] |
| Alendronate_Alfacalcidol:Clodronate | 0 | Major concerns | Low risk | Some concerns | Some concerns | No concerns | Some concerns | Low | ["Within-study bias"] |
| Alendronate_Alfacalcidol:Denosumab | 0 | Some concerns | Low risk | No concerns | No concerns | Some concerns | Some concerns | Moderate | - |
| Alendronate_Alfacalcidol:Etidronate | 0 | Major concerns | Low risk | Some concerns | No concerns | No concerns | Some concerns | Low | ["Within-study bias"] |
| Alendronate_Alfacalcidol:Raloxifene | 0 | Some concerns | Low risk | Some concerns | No concerns | No concerns | Some concerns | Moderate | - |
| Alendronate_Alfacalcidol:Risedronate | 0 | Some concerns | Low risk | No concerns | Some concerns | No concerns | Some concerns | Moderate | - |
| Alendronate_Alfacalcidol:Teriparatide | 0 | Major concerns | Low risk | Some concerns | No concerns | Some concerns | Some concerns | Low | ["Within-study bias"] |
| Alendronate_Alfacalcidol:Teriparatide+Alendronate | 0 | Major concerns | Low risk | Some concerns | Some concerns | No concerns | Some concerns | Low | ["Within-study bias"] |
| Alendronate_Alfacalcidol:Zoledronate | 0 | Some concerns | Low risk | Some concerns | No concerns | No concerns | Some concerns | Moderate | - |
| Alfacalcidol:Clodronate | 0 | Some concerns | Low risk | No concerns | No concerns | No concerns | Some concerns | Moderate | - |
| Alfacalcidol:Denosumab | 0 | Some concerns | Low risk | No concerns | No concerns | No concerns | Some concerns | Moderate | - |
| Alfacalcidol:Etidronate | 0 | Some concerns | Low risk | No concerns | No concerns | Some concerns | Some concerns | Moderate | - |
| Alfacalcidol:Raloxifene | 0 | Some concerns | Low risk | No concerns | Some concerns | No concerns | Some concerns | Moderate | - |
| Alfacalcidol:Risedronate | 0 | Some concerns | Low risk | No concerns | No concerns | No concerns | Some concerns | Moderate | - |
| Alfacalcidol:Teriparatide | 0 | Some concerns | Low risk | Some concerns | No concerns | Some concerns | Some concerns | Moderate | - |
| Alfacalcidol:Teriparatide+Alendronate | 0 | Some concerns | Low risk | Some concerns | No concerns | No concerns | Some concerns | Moderate | - |
| Alfacalcidol:Zoledronate | 0 | Some concerns | Low risk | No concerns | No concerns | Some concerns | Some concerns | Moderate | - |
| Clodronate:Denosumab | 0 | Some concerns | Low risk | No concerns | No concerns | Some concerns | Some concerns | Moderate | - |
| Clodronate:Etidronate | 0 | Some concerns | Low risk | No concerns | No concerns | Some concerns | Some concerns | Moderate | - |
| Clodronate:Raloxifene | 0 | Some concerns | Low risk | No concerns | Some concerns | No concerns | Some concerns | Moderate | - |
| Clodronate:Risedronate | 0 | Some concerns | Low risk | No concerns | No concerns | No concerns | Some concerns | High | - |
| Clodronate:Teriparatide | 0 | Some concerns | Low risk | Some concerns | Some concerns | No concerns | Some concerns | Moderate | - |
| Clodronate:Teriparatide+Alendronate | 0 | Some concerns | Low risk | Some concerns | Some concerns | No concerns | Some concerns | Moderate | - |
| Clodronate:Zoledronate | 0 | Some concerns | Low risk | No concerns | No concerns | Some concerns | Some concerns | Moderate | - |
| Denosumab:Etidronate | 0 | Some concerns | Low risk | No concerns | No concerns | No concerns | Some concerns | High | - |
| Denosumab:Raloxifene | 0 | No concerns | Low risk | No concerns | No concerns | Some concerns | Some concerns | High | - |
| Denosumab:Teriparatide | 0 | Some concerns | Low risk | No concerns | No concerns | No concerns | Some concerns | High | - |
| Denosumab:Teriparatide+Alendronate | 0 | Some concerns | Low risk | No concerns | No concerns | No concerns | Some concerns | High | - |
| Denosumab:Zoledronate | 0 | Some concerns | Low risk | No concerns | No concerns | No concerns | Some concerns | High | - |
| Etidronate:Raloxifene | 0 | Some concerns | Low risk | No concerns | No concerns | No concerns | Some concerns | High | - |
| Etidronate:Risedronate | 0 | Some concerns | Low risk | No concerns | No concerns | Some concerns | Some concerns | Moderate | - |
| Etidronate:Teriparatide | 0 | Some concerns | Low risk | Some concerns | No concerns | Some concerns | Some concerns | Moderate | - |
| Etidronate:Teriparatide+Alendronate | 0 | Some concerns | Low risk | Some concerns | No concerns | Some concerns | Some concerns | Moderate | - |
| Etidronate:Zoledronate | 0 | Some concerns | Low risk | No concerns | No concerns | No concerns | Some concerns | High | - |
| Raloxifene:Risedronate | 0 | No concerns | Low risk | No concerns | No concerns | Some concerns | Some concerns | High | - |
| Raloxifene:Teriparatide | 0 | Some concerns | Low risk | Some concerns | No concerns | Some concerns | Some concerns | High | - |
| Raloxifene:Teriparatide+Alendronate | 0 | Some concerns | Low risk | Some concerns | Some concerns | No concerns | Some concerns | Moderate | - |
| Raloxifene:Zoledronate | 0 | No concerns | Low risk | No concerns | No concerns | No concerns | Some concerns | High | - |
| Risedronate:Teriparatide | 0 | Some concerns | Low risk | No concerns | No concerns | No concerns | Some concerns | High | - |
| Risedronate:Teriparatide+Alendronate | 0 | Some concerns | Low risk | No concerns | No concerns | No concerns | Some concerns | High | - |
| Risedronate:Zoledronate | 0 | No concerns | Low risk | No concerns | No concerns | Some concerns | Some concerns | High | - |
| Teriparatide:Teriparatide+Alendronate | 0 | Some concerns | Low risk | Some concerns | No concerns | No concerns | Some concerns | Moderate | - |
| Teriparatide:Zoledronate | 0 | Some concerns | Low risk | Some concerns | No concerns | No concerns | Some concerns | Moderate | - |
| Teriparatide+Alendronate:Zoledronate | 0 | Some concerns | Low risk | Some concerns | No concerns | Some concerns | Some concerns | Moderate | - |

**Table 8.3 CINeMA report (24 months)**

| Comparison | Number of studies | Within-study bias | Reporting bias | Indirectness | Imprecision | Heterogeneity | Incoherence | Confidence rating | Reason(s) for downgrading |
| --- | --- | --- | --- | --- | --- | --- | --- | --- | --- |
| Alendronate:Alfacalcidol | 1 | Some concerns | Low risk | Some concerns | No concerns | No concerns | No concerns | Moderate | - |
| Alendronate:Placebo | 4 | Some concerns | Low risk | Some concerns | No concerns | No concerns | No concerns | Moderate | - |
| Alendronate:Teriparatide+Alendronate | 1 | Some concerns | Low risk | Some concerns | No concerns | No concerns | No concerns | Moderate | - |
| Alfacalcidol:Placebo | 1 | Some concerns | Low risk | Some concerns | No concerns | No concerns | No concerns | Moderate | - |
| Denosumab:Risedronate | 1 | Some concerns | Low risk | No concerns | No concerns | No concerns | No concerns | High | - |
| Etidronate:Placebo | 1 | Some concerns | Low risk | No concerns | No concerns | No concerns | No concerns | High | - |
| Placebo:Raloxifene | 1 | No concerns | Low risk | No concerns | No concerns | No concerns | No concerns | High | - |
| Placebo:Risedronate | 1 | No concerns | Low risk | No concerns | No concerns | No concerns | No concerns | High | - |
| Placebo:Teriparatide+Alendronate | 1 | Some concerns | Low risk | Some concerns | No concerns | Some concerns | No concerns | Moderate | - |
| Placebo:Zoledronate | 3 | Some concerns | Low risk | No concerns | No concerns | No concerns | No concerns | High | - |
| Alendronate:Denosumab | 0 | Some concerns | Low risk | No concerns | No concerns | No concerns | No concerns | High | - |
| Alendronate:Etidronate | 0 | Some concerns | Low risk | No concerns | No concerns | No concerns | No concerns | High | - |
| Alendronate:Raloxifene | 0 | Some concerns | Low risk | No concerns | No concerns | No concerns | No concerns | High | - |
| Alendronate:Risedronate | 0 | Some concerns | Low risk | No concerns | No concerns | No concerns | No concerns | High | - |
| Alendronate:Zoledronate | 0 | Some concerns | Low risk | No concerns | No concerns | No concerns | No concerns | High | - |
| Alfacalcidol:Denosumab | 0 | Some concerns | Low risk | No concerns | No concerns | No concerns | No concerns | High | - |
| Alfacalcidol:Etidronate | 0 | Some concerns | Low risk | Some concerns | No concerns | No concerns | No concerns | Moderate | - |
| Alfacalcidol:Raloxifene | 0 | Some concerns | Low risk | Some concerns | No concerns | Some concerns | No concerns | Moderate | - |
| Alfacalcidol:Risedronate | 0 | Some concerns | Low risk | Some concerns | No concerns | No concerns | No concerns | Moderate | - |
| Alfacalcidol:Teriparatide+Alendronate | 0 | Some concerns | Low risk | Some concerns | No concerns | No concerns | No concerns | Moderate | - |
| Alfacalcidol:Zoledronate | 0 | Some concerns | Low risk | Some concerns | No concerns | No concerns | No concerns | Moderate | - |
| Denosumab:Etidronate | 0 | Some concerns | Low risk | No concerns | No concerns | No concerns | No concerns | High | - |
| Denosumab:Placebo | 0 | Some concerns | Low risk | No concerns | No concerns | No concerns | No concerns | High | - |
| Denosumab:Raloxifene | 0 | No concerns | Low risk | No concerns | No concerns | No concerns | No concerns | High | - |
| Denosumab:Teriparatide+Alendronate | 0 | Some concerns | Low risk | No concerns | No concerns | Some concerns | No concerns | Moderate | - |
| Denosumab:Zoledronate | 0 | Some concerns | Low risk | No concerns | No concerns | Some concerns | No concerns | Moderate | - |
| Etidronate:Raloxifene | 0 | Some concerns | Low risk | No concerns | No concerns | Some concerns | No concerns | Moderate | - |
| Etidronate:Risedronate | 0 | Some concerns | Low risk | No concerns | No concerns | No concerns | No concerns | High | - |
| Etidronate:Teriparatide+Alendronate | 0 | Some concerns | Low risk | Some concerns | No concerns | No concerns | No concerns | Moderate | - |
| Etidronate:Zoledronate | 0 | Some concerns | Low risk | No concerns | No concerns | No concerns | No concerns | High | - |
| Raloxifene:Risedronate | 0 | No concerns | Low risk | No concerns | No concerns | No concerns | No concerns | High | - |
| Raloxifene:Teriparatide+Alendronate | 0 | Some concerns | Low risk | Some concerns | No concerns | No concerns | No concerns | Moderate | - |
| Raloxifene:Zoledronate | 0 | No concerns | Low risk | No concerns | No concerns | No concerns | No concerns | High | - |
| Risedronate:Teriparatide+Alendronate | 0 | Some concerns | Low risk | Some concerns | No concerns | Some concerns | No concerns | Moderate | - |
| Risedronate:Zoledronate | 0 | No concerns | Low risk | No concerns | No concerns | No concerns | No concerns | High | - |
| Teriparatide+Alendronate:Zoledronate | 0 | Some concerns | Low risk | Some concerns | No concerns | No concerns | No concerns | Moderate | - |

# Appendix 9: Additional analyses

**Figure 9.1 Network plots for additional analyses comparing (6 months)**


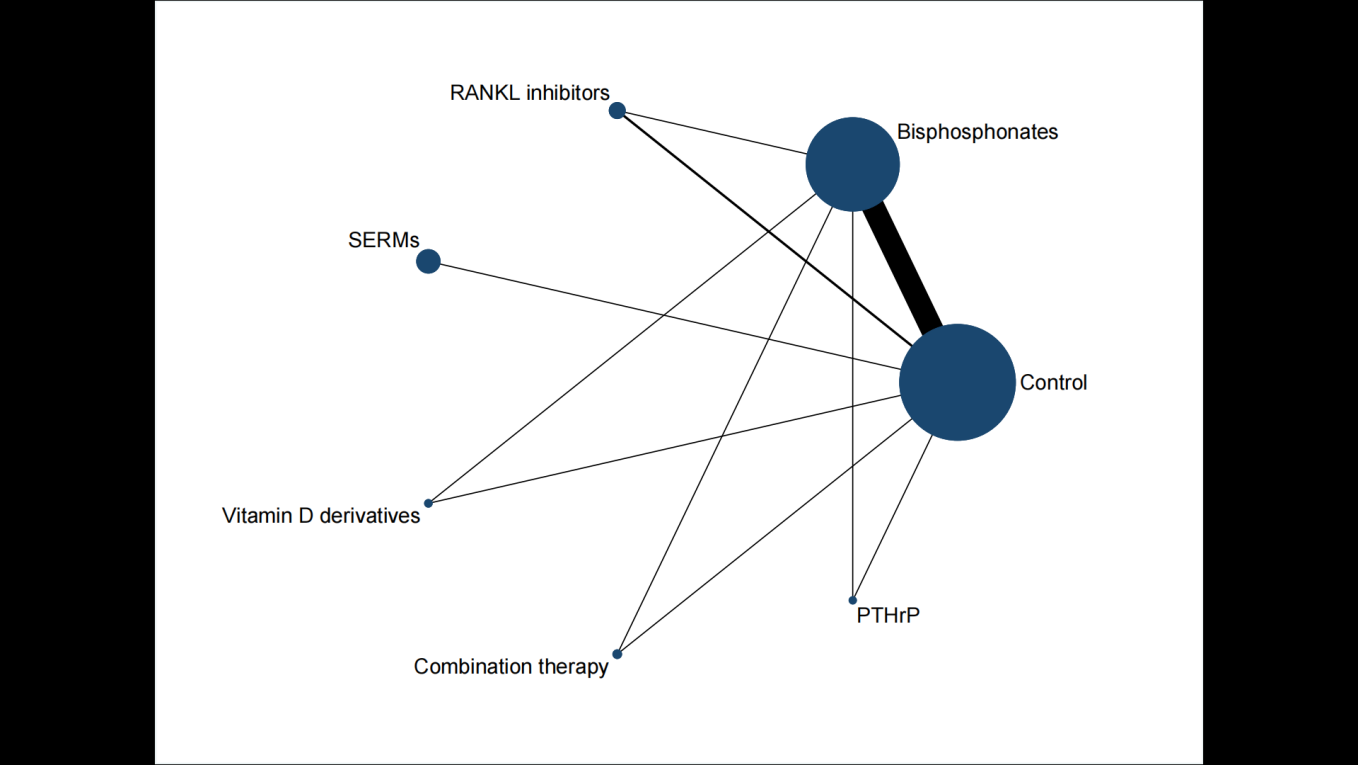


**Figure 9.2 Network plots for additional analyses comparing (12 months)**


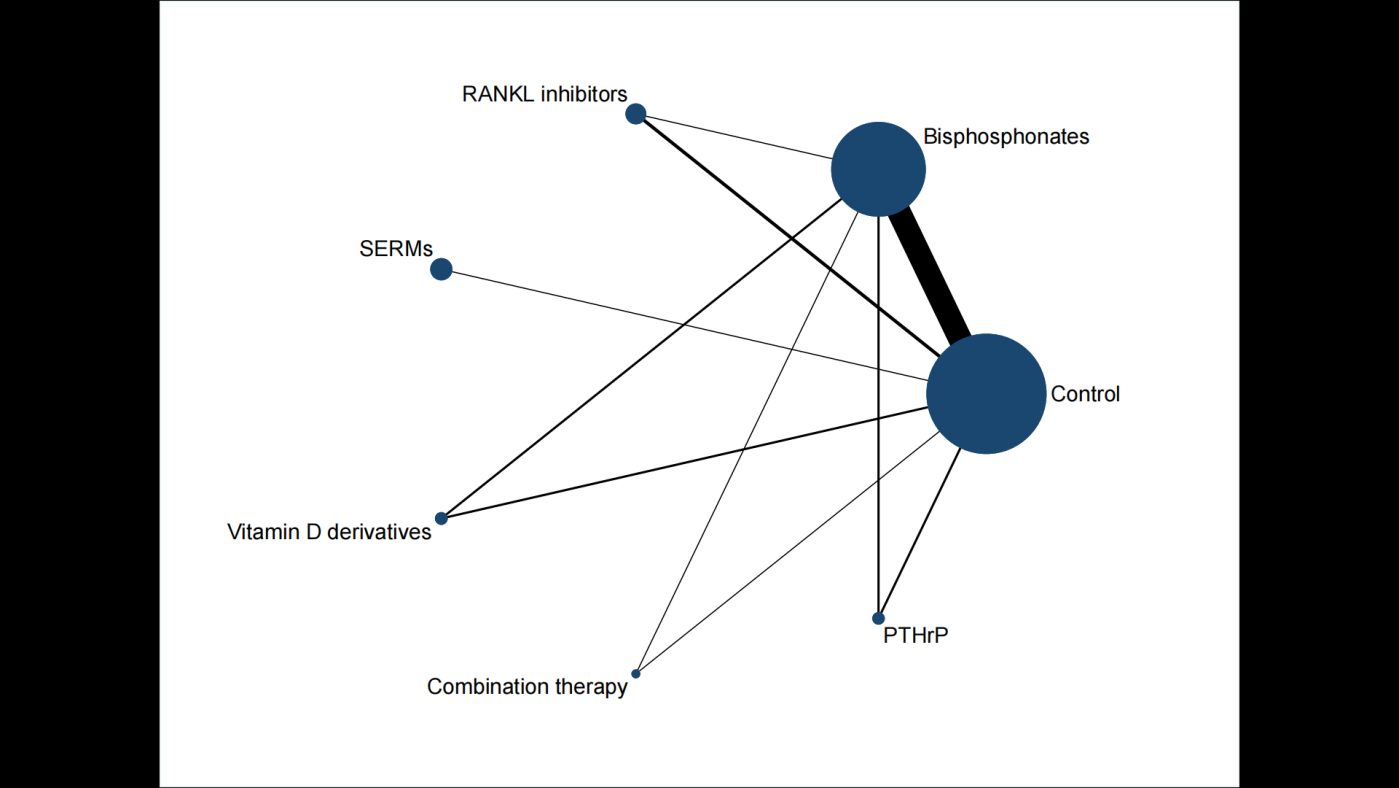


**Figure 9.3 Network plots for additional analyses comparing (24 months and over)**


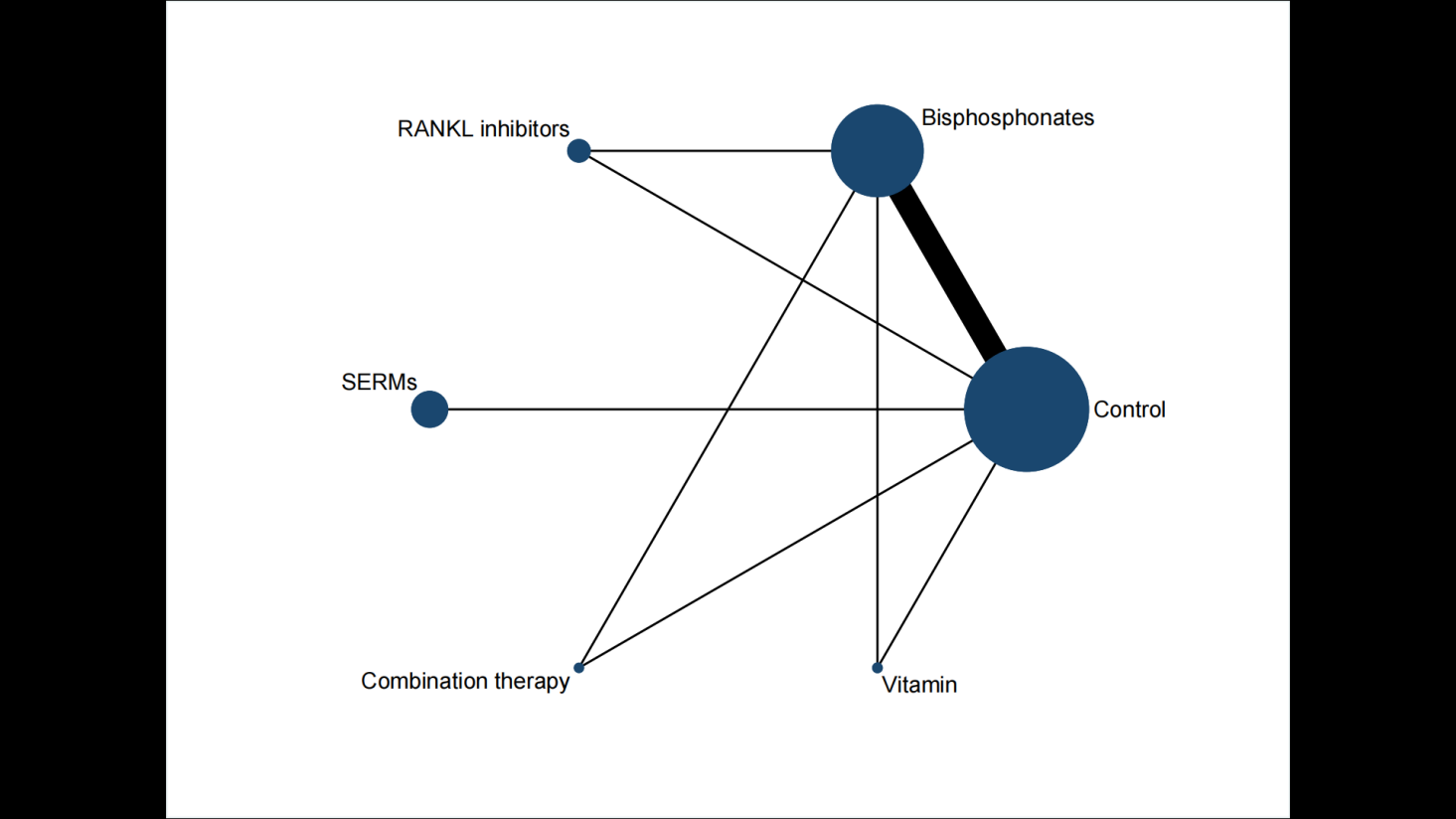


**Figure 9.4 Forest plot for additional analyses (6 months)**


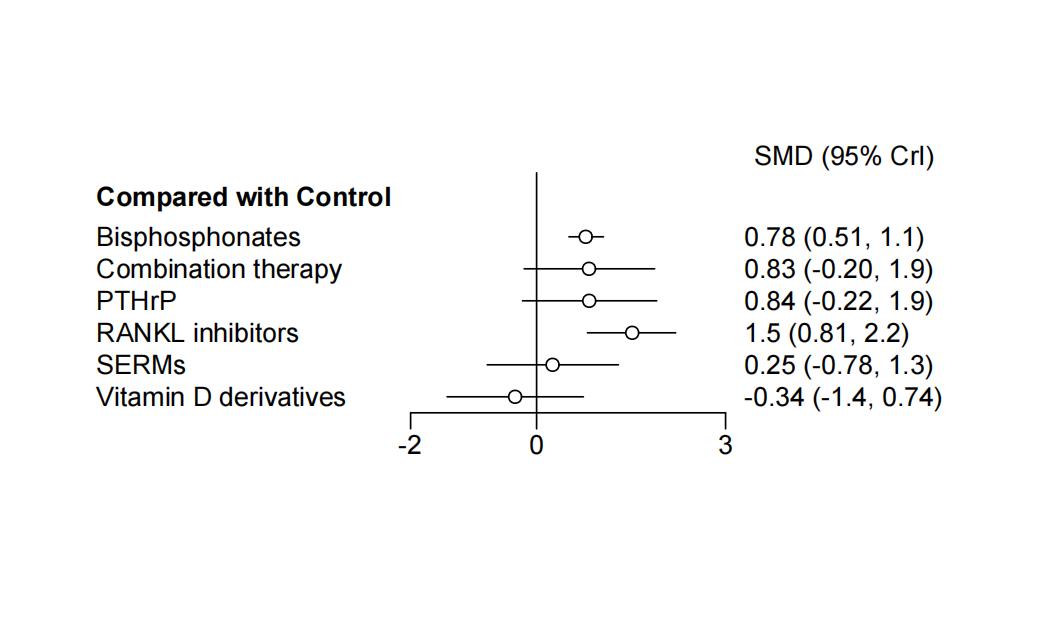


**Figure 9.5 Forest plot for additional analyses (12 months)**


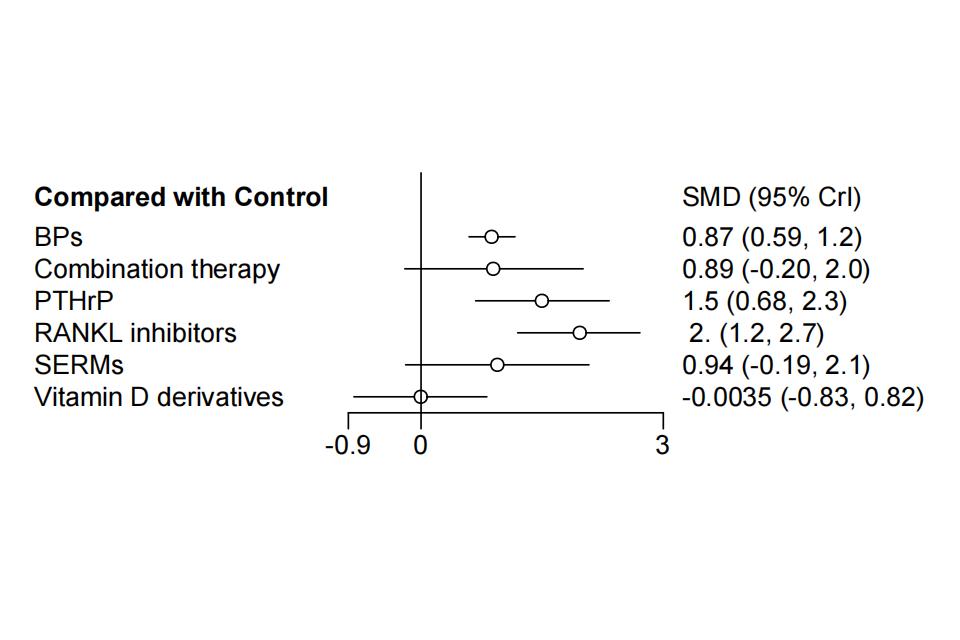


**Figure 9.6 Forest plot for additional analyses (24 month and overs)**


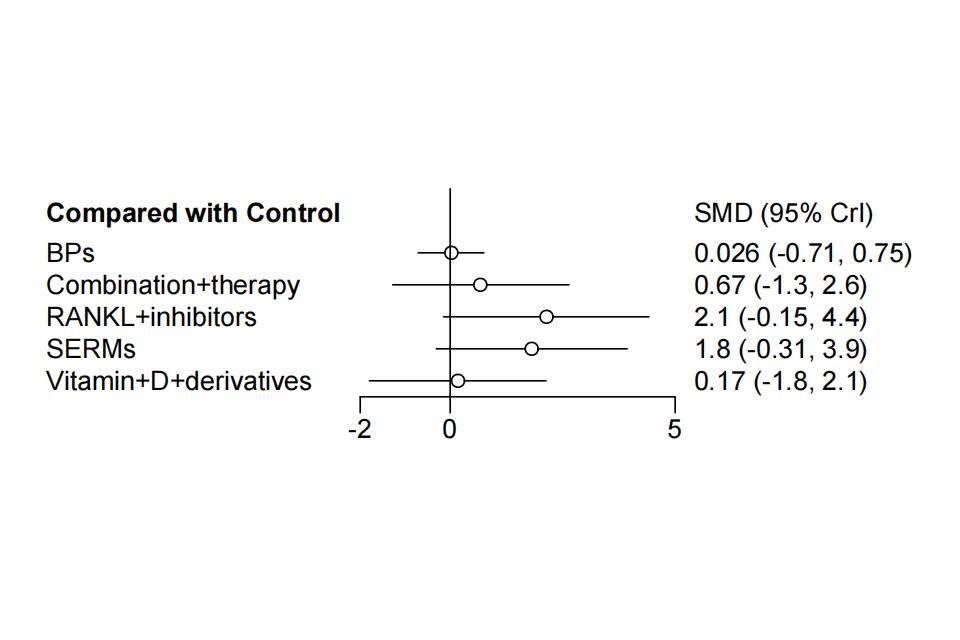


**Table 9.1 SUCRA of prophylactic efficacy on BMD in calcar (additional analyses)**

|  | 6months | 12months | 24months and above |
| --- | --- | --- | --- |
| Bisphosphonates | 0.63 | 0.50 | 0.26 |
| Combination therapy | 0.64 | 0.51 | 0.51 |
| Control | 0.19 | 0.10 | 0.24 |
| PTHrP | 0.64 | 0.79 | NA |
| RANKL inhibitors | 0.95 | 0.95 | 0.86 |
| SERMs | 0.35 | 0.54 | 0.80 |
| Vitamin D derivatives | 0.10 | 0.12 | 0.33 |
| BMD, bone mineral density; NA, not applicable; SUCRA, surface under cumulative ranking curve. | | | |
|  |  |  |  |

**Appendix 10 PRISMA NMA Checklist of Items to Include When Reporting A Systematic Review Involving a Network Meta-analysis**

| **Section/Topic** | **Item #** | **Checklist Item** | **Reported on Page #** |
| --- | --- | --- | --- |
| **TITLE** |  |  |  |
| Title | 1 | Identify the report as a systematic review *incorporating a network meta-analysis (or related form of meta-analysis).* | ***#1*** |
|  |  |  |  |
| **ABSTRACT** |  |  |  |
| Structured summary | 2 | Provide a structured summary including, as applicable:  **Background:** main objectives  **Methods:** data sources; study eligibility criteria, participants, and interventions; study appraisal; and *synthesis methods, such as network meta-analysis.*  **Results:** number of studies and participants identified; summary estimates with corresponding confidence/credible intervals; *treatment rankings may also be discussed. Authors may choose to summarize pairwise comparisons against a chosen treatment included in their analyses for brevity.*  **Discussion/Conclusions:** limitations; conclusions and implications of findings.  **Other:** primary source of funding; systematic review registration number with registry name. | #1 |
|  |  |  |  |
| **INTRODUCTION** |  |  |  |
| Rationale | 3 | Describe the rationale for the review in the context of what is already known*, including mention of why a network meta-analysis has been conducted.* | ***#2*** |
| Objectives | 4 | Provide an explicit statement of questions being addressed, with reference to participants, interventions, comparisons, outcomes, and study design (PICOS). | #2 |
|  |  |  |  |
| **METHODS** |  |  |  |
| Protocol and registration | 5 | Indicate whether a review protocol exists and if and where it can be accessed (e.g., Web address); and, if available, provide registration information, including registration number. | #2 |
| Eligibility criteria | 6 | Specify study characteristics (e.g., PICOS, length of follow-up) and report characteristics (e.g., years considered, language, publication status) used as criteria for eligibility, giving rationale. *Clearly describe eligible treatments included in the treatment network, and note whether any have been clustered or merged into the same node (with justification).* | ***#3*** |
| Information sources | 7 | Describe all information sources (e.g., databases with dates of coverage, contact with study authors to identify additional studies) in the search and date last searched. | #3 |
| Search | 8 | Present full electronic search strategy for at least one database, including any limits used, such that it could be repeated. | Appendix1 |
| Study selection | 9 | State the process for selecting studies (i.e., screening, eligibility, included in systematic review, and, if applicable, included in the meta-analysis). | #4 |
| Data collection process | 10 | Describe method of data extraction from reports (e.g., piloted forms, independently, in duplicate) and any processes for obtaining and confirming data from investigators. | #4 |
| Data items | 11 | List and define all variables for which data were sought (e.g., PICOS, funding sources) and any assumptions and simplifications made. | #4 |
| **Geometry of the network** | **S1** | Describe methods used to explore the geometry of the treatment network under study and potential biases related to it. This should include how the evidence base has been graphically summarized for presentation, and what characteristics were compiled and used to describe the evidence base to readers. | ***#5*** |
| Risk of bias within individual studies | 12 | Describe methods used for assessing risk of bias of individual studies (including specification of whether this was done at the study or outcome level), and how this information is to be used in any data synthesis. | #5 |
| Summary measures | 13 | State the principal summary measures (e.g., risk ratio, difference in means). *Also describe the use of additional summary measures assessed, such as treatment rankings and surface under the cumulative ranking curve (SUCRA) values, as well as modified approaches used to present summary findings from meta-analyses.* | #5 |
| Planned methods of analysis | 14 | Describe the methods of handling data and combining results of studies for each network meta-analysis. This should include, but not be limited to:   - *Handling of multi-arm trials;* - *Selection of variance structure;* - *Selection of prior distributions in Bayesian analyses; and* - *Assessment of model fit.* | #5，#6 |
| **Assessment of Inconsistency** | **S2** | Describe the statistical methods used to evaluate the agreement of direct and indirect evidence in the treatment network(s) studied. Describe efforts taken to address its presence when found. | #5，#6 |
| Risk of bias across studies | 15 | Specify any assessment of risk of bias that may affect the cumulative evidence (e.g., publication bias, selective reporting within studies). | #5，#6 |
| Additional analyses | 16 | Describe methods of additional analyses if done, indicating which were pre-specified. This may include, but not be limited to, the following:   - Sensitivity or subgroup analyses; - Meta-regression analyses; - *Alternative formulations of the treatment network; and* - *Use of alternative prior distributions for Bayesian analyses (if applicable).* | #6 |
|  |  |  |  |
| **RESULTS†** |  |  |  |
| Study selection | 17 | Give numbers of studies screened, assessed for eligibility, and included in the review, with reasons for exclusions at each stage, ideally with a flow diagram. | #6 |
| **Presentation of network structure** | **S3** | Provide a network graph of the included studies to enable visualization of the geometry of the treatment network. | #6 |
| **Summary of network geometry** | **S4** | Provide a brief overview of characteristics of the treatment network. This may include commentary on the abundance of trials and randomized patients for the different interventions and pairwise comparisons in the network, gaps of evidence in the treatment network, and potential biases reflected by the network structure. | ***#6*** |
| Study characteristics | 18 | For each study, present characteristics for which data were extracted (e.g., study size, PICOS, follow-up period) and provide the citations. | #6 |
| Risk of bias within studies | 19 | Present data on risk of bias of each study and, if available, any outcome level assessment. | Appendix 3 |
| Results of individual studies | 20 | For all outcomes considered (benefits or harms), present, for each study: 1) simple summary data for each intervention group, and 2) effect estimates and confidence intervals. *Modified approaches may be needed to deal with information from larger networks.* | Appendix 5 |
| Synthesis of results | 21 | Present results of each meta-analysis done, including confidence/credible intervals. *In larger networks, authors may focus on comparisons versus a particular comparator (e.g. placebo or standard care), with full findings presented in an appendix. League tables and forest plots may be considered to summarize pairwise comparisons.* If additional summary measures were explored (such as treatment rankings), these should also be presented. | #6,#7  Appendix 5 |
| **Exploration for inconsistency** | **S5** | Describe results from investigations of inconsistency. This may include such information as measures of model fit to compare consistency and inconsistency models, *P* values from statistical tests, or summary of inconsistency estimates from different parts of the treatment network. | ***#7,#8***  Appendix 6 |
| Risk of bias across studies | 22 | Present results of any assessment of risk of bias across studies for the evidence base being studied. | ***#7,#8***  Appendix 7  Appendix 8 |
| Results of additional analyses | 23 | Give results of additional analyses, if done (e.g., sensitivity or subgroup analyses, meta-regression analyses*, alternative network geometries studied, alternative choice of prior distributions for Bayesian analyses,* and so forth). | ***#8***  Appendix 9 |
|  |  |  |  |
| **DISCUSSION** |  |  |  |
| Summary of evidence | 24 | Summarize the main findings, including the strength of evidence for each main outcome; consider their relevance to key groups (e.g., healthcare providers, users, and policy-makers). | #8-#12 |
| Limitations | 25 | Discuss limitations at study and outcome level (e.g., risk of bias), and at review level (e.g., incomplete retrieval of identified research, reporting bias). *Comment on the validity of the assumptions, such as transitivity and consistency. Comment on any concerns regarding network geometry (e.g., avoidance of certain comparisons).* | #12 |
| Conclusions | 26 | Provide a general interpretation of the results in the context of other evidence, and implications for future research. | #12 |
|  |  |  |  |
| **FUNDING** |  |  |  |
| Funding | 27 | Describe sources of funding for the systematic review and other support (e.g., supply of data); role of funders for the systematic review. This should also include information regarding whether funding has been received from manufacturers of treatments in the network and/or whether some of the authors are content experts with professional conflicts of interest that could affect use of treatments in the network. |  |

PICOS = population, intervention, comparators, outcomes, study design.

* Text in italics indicateS wording specific to reporting of network meta-analyses that has been added to guidance from the PRISMA statement.

† Authors may wish to plan for use of appendices to present all relevant information in full detail for items in this section.
